# Human disturbances affect the topology of food webs

**DOI:** 10.1111/ele.14107

**Published:** 2022-09-27

**Authors:** Frederico Mestre, Alejandro Rozenfeld, Miguel B. Araújo

**Affiliations:** ^1^ ‘Rui Nabeiro’ Biodiversity Chair, MED – Mediterranean Institute for Agriculture, Environment and Development & CHANGE – Global Change and Sustainability Institute, Institute for Advanced Studies and Research Universidade de Évora Évora Portugal; ^2^ Centro de Investigaciones en Física e Ingeniería del Centro Universidad Nacional del Centro de la Provincia de Buenos Aires, Consejo Nacional de Investigaciones Científicas y Técnicas Tandil Buenos Aires Argentina; ^3^ CONICET‐CIFICEN‐Universidad del Centro de la Provincia de Buenos Aires Tandil Buenos Aires Argentina; ^4^ Department of Biogeography and Global Change, National Museum of Natural Sciences CSIC Madrid Spain

**Keywords:** anthropogenic disturbance, ecosystem functioning, network topology, trophic interactions

## Abstract

Networks describe nodes connected by links, with numbers of links per node, the degree, forming a range of distributions including random and scale‐free. How network topologies emerge in natural systems still puzzles scientists. Based on previous theoretical simulations, we predict that scale‐free food webs are favourably selected by random disturbances while random food webs are selected by targeted disturbances. We assume that lower human pressures are more likely associated with random disturbances, whereas higher pressures are associated with targeted ones. We examine these predictions using 351 empirical food webs, generally confirming our predictions. Should the topology of food webs respond to changes in the magnitude of disturbances in a predictable fashion, consistently across ecosystems and scales of organisation, it would provide a baseline expectation to understand and predict the consequences of human pressures on ecosystem dynamics.

## Graphical abstract

We predict that food webs with scale‐free degree distributions should be favourably selected by random disturbances (in regions less disturbed by human pressure) while food webs with random degree distributions would be associated with targeted disturbances (in areas with more human pressure). We examine our predictions using 351 empirical food webs and confirmed that random food webs were more common in areas exposed to higher levels of human pressure, while scale‐free food webs prevailed where pressures were lower.

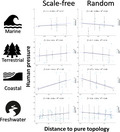

## INTRODUCTION

Food webs characterise fluxes of energy and matter throughout ecosystems, being a fundamental expression of ecosystem functioning (Barnes et al., 2018) and one of the more frequently studied types of ecological networks (Morales‐Castilla et al., 2015). The structure of food webs (Camacho et al., 2002; Dunne et al., 2002a; Williams & Martinez, 2000) and how they respond to environmental gradients and human disturbances (Ings et al., 2009; Pellissier et al., 2017; Thompson et al., 2012; Tylianakis & Morris, 2017) has been extensively studied and few generalities have emerged or agreed upon (Mestre et al., 2022). While trophic structures have been shown to vary across environmental or human‐related gradients (Albouy et al., 2019; Kortsch et al., 2019; Layer et al., 2010; Mendoza & Araújo, 2019, 2022), there still is a debate as to whether such drivers would affect metrics of network topology among empirical food webs, how these metrics would be affected and how much (Dunne et al., 2002a).

Previous theoretical simulations enabled a few predictions on these topics, but beg for empirical testing. An influential study based on simulations of attacks across artificially constructed networks (Albert et al., 2000) proposed that networks with different distributions of links among nodes (different degree distribution) would be differently affected by distinct types of attacks. Targeted attacks on networks should disproportionately affect those with a scale‐free degree distribution. In contrast, networks with random degree distribution should be less sensitive to targeted attacks.

It follows from these simulations that, in dynamic adaptive systems, networks affected by different types of disturbances (random or targeted), should select favourably networks with different topologies. Inspired by these simulations, we propose that, all other things being equal, ecosystems exposed to random disturbances would favourably select networks with scale‐free degree distributions, whereas ecosystems exposed to non‐random disturbances would favourably select networks with random degree distribution.

Categorising disturbances as random or targeted is difficult outside the realm of controlled simulations though. To address the issue, we make the following assumptions. First, random disturbances predominate in ecosystems exposed to stochastic dynamics of local extinction and colonisation, as one would expect if populations were subject to background natural variability (Hanski, 1991, 1998; Keymer et al., 2000). Second, targeted disturbances predominate in ecosystems exposed to extrinsic disturbances to natural population dynamics. That is the case of areas exposed to high levels of human‐driven disturbances, which unlike natural stochasticity are geographically structured. The assumption is supported by evidence that human disturbances induce spatially or phylogenetically clustered patterns of threat (Safi & Pettorelli, 2010), targeting species with poor dispersal ability, slow life histories, large body sizes, or narrow habitat breadth (Chichorro et al., 2020; González‐Suárez et al., 2013; Lee & Jetz, 2011; Purvis et al., 2000; Suraci et al., 2021).

We expect a consistent trend for stochastic network node removal across low disturbance regions, while targeted node removal should predominate across regions exposed to high levels of extrinsic disturbances. Given that targeted disturbances arising from human impacts are exceptional and overlap with background disturbances, any deviation from the expected topology should be captured by variation in targeted disturbances caused by human impacts rather than by random processes.

**FIGURE 1:**
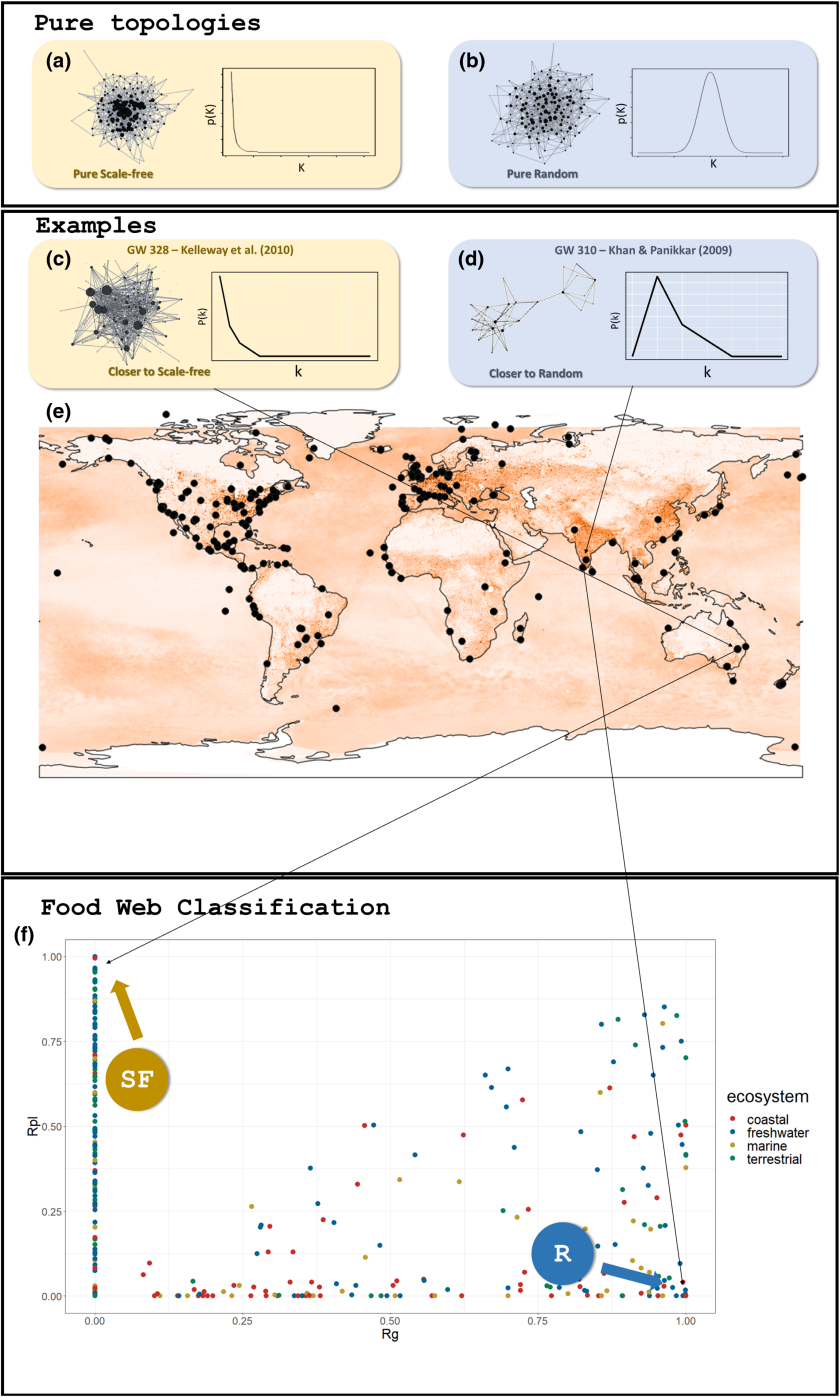
Typical network configuration and degree distribution in each one of the topologies considered (top panel), empirical examples from the dataset, geographical distribution and human pressure (mid panel) and food web classification (bottom panel): (a) pure scale‐free network (network and degree distribution) topology; (b) pure random network topology; (c) example of a network close to pure scale‐free topology (Kelleway et al., 2010); (d) example of a network close to pure random topology (Khan & Panikkar, 2009); (e) global distribution of the food webs in the dataset with the terrestrial impact metric (human footprint [Venter et al., 2016a, 2016b]) and the marine impact metric (cumulative impact to marine ecosystems (Halpern et al., 2015)) scaled from 0 to 1 for comparability; (f) categorisation of the food webs in the dataset where the signalled corners are those in which the food webs are closer to each of the two pure topologies (*x* axis: Correlation coefficient to gaussian (*R*_G_); *y* axis: Correlation coefficient to power‐law (*R*_PL_); see methods). The pattern observed in panel (f), whereby some values fall along the y axis (RG = 0) and almost all points fall below an imagined diagonal line (defined RG = RPL), is a consequence of Equation 2. According to this equation, the computation of RG is conducted only for food webs to which the degree distribution is such that the left portion of the curve is present (μ + σ/3 > 0), and the maximum degree (Kmax) is big enough to also allow the right portion of the curve to be present (kmax > μ + σ) (Figure S5). Food webs with degree distributions not conforming to these conditions would have an RG = 0, which is the case with most that would otherwise occupy the upper triangle of the plot.

**FIGURE 2:**
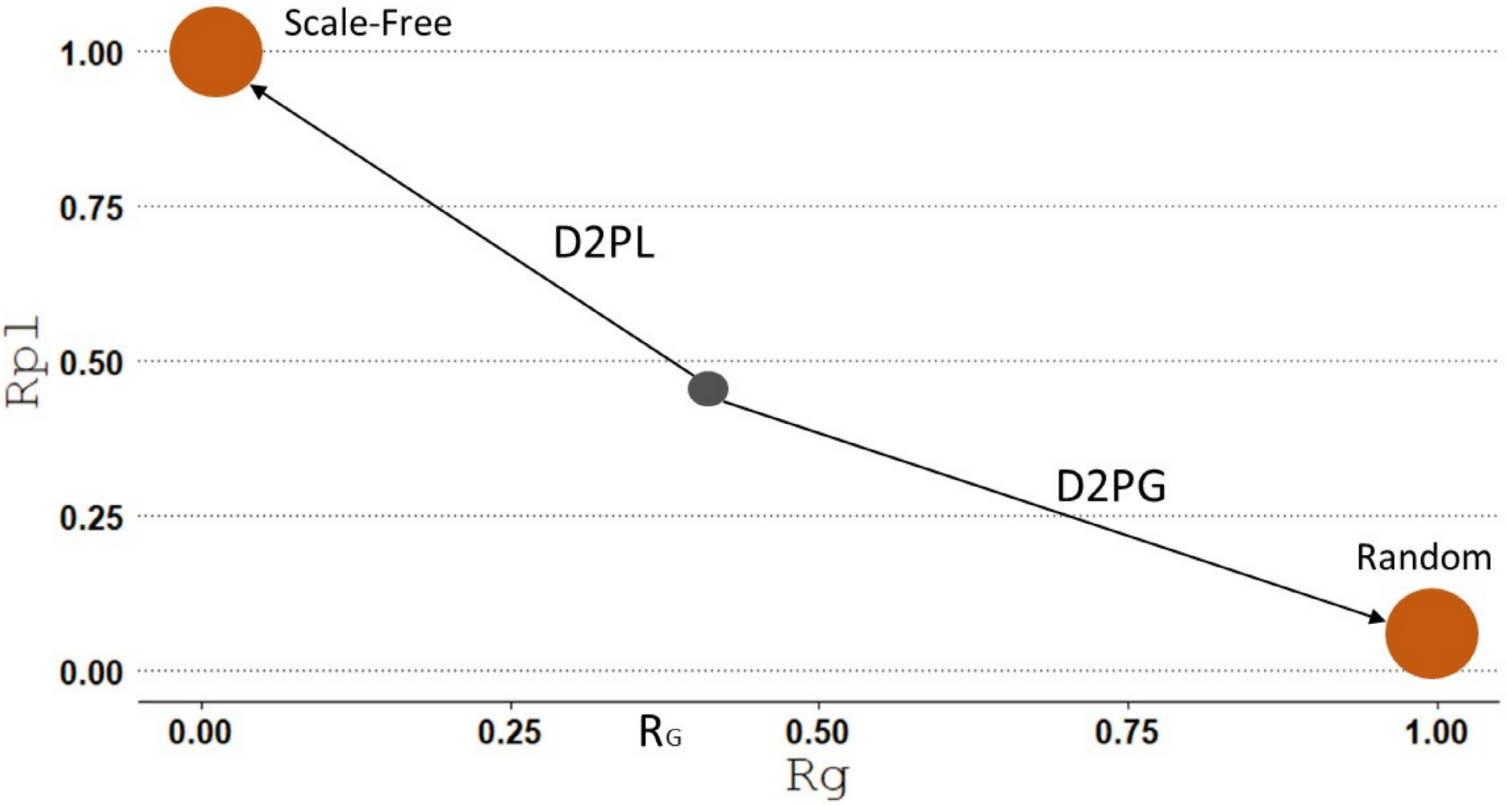
Location of an hypothetical food web in the (*R*_G_, R_PL_) plane (grey circle), with the distances to pure gaussian (random; D2PG) and pure power‐law (D2PL) characterising its topology.

One difficulty with testing theoretical inferences of food web topology at broad geographical scales is that empirical food web data are under‐replicated, noisy, gathered for a variety of purposes and using a diversity of methods (Mestre et al., 2022). Consequently, generalisations with such data are difficult. To address under‐replication, we gathered a globally distributed multiple ecosystem food web database (Figure 1, panel e). To maximise comparability across databases, we pruned the dataset with several exclusion rules (see methods). To avoid imposing a ‘black and white’ topological classification on networks that would neglect the actual fuzziness in the data, we determined the degree distribution of each food web and then developed an approach that measures their distance to pure scale‐free and random topologies (Figure 1, panel f; Figure 2). Next, we evaluated the relationship between the distance to pure topologies of degree distribution and the level of human disturbance after matching the food web location with a human disturbance index (for the ocean Halpern et al., 2015; for land, coastlines and freshwater Venter et al., 2016a, 2016b). To reduce noise, typical of large‐scale ecological data collected for a variety of different purposes, we binned the food web data. Binning is a familiar approach in data mining to help elucidate relationships obscured by noisy data (Han et al., 2012; Pyle, 1999). Finally, we simulated extinctions (Bellingeri et al., 2013) to compare the robustness to extinction of food webs with varying distances to pure random and pure scale‐free topologies.

## MATERIAL AND METHODS

### Food web and anthropogenic impact datasets

Food web data were retrieved from online databases, the Globalweb (Thompson et al., 2012) and EcoBase (Colléter et al., 2013). We obtained 393 food web matrices and kept 351, after removing those with one or more non‐numerical values, repeated species names and absence of accurate information on the geographical location (Appendix S3). For food webs obtained from Globalweb, we retrieved the geographical location from the original publication. The dataset used has a global coverage (Figure 1, panel e) and encompasses data across the four general types of ecosystems on earth, that is, coastal (29.34%), freshwater (36.47%), marine (15.10%) and terrestrial (19.09%) (Figure S1). The food webs ranged, in number of nodes, from 3 to 162 (first quartile = 13; median = 21; mean = 29.18; third quartile = 33) and, in number of links, from 2 to 1902 (first quartile = 26; median = 62; mean = 114.7; third quartile = 141.5). Given the diversity of food web data sources, there is substantial heterogeneity in the resolution of nodes (both at taxonomic, e.g. species vs. higher‐order classifications, and functional levels, e.g. empirically derived vs. inferred trophic relationships). Different resolutions across (and within) food webs can affect the topology thus the comparability among them (Hemprich‐Bennett et al., 2021). This is a common shortcoming in studies resorting to pooled data sets, such as the Globalweb database; they generally have to live with and consider the data limitations carefully when interpreting results (e.g. Baiser et al., 2019; Mora et al., 2018). The a priori expectation is that a large number of food webs pooled together, their global distribution, and the wide coverage of ecosystem types encompassed, contributes to smooth away sources of noise and allows revealing emergent signals arising from the data.

Information on human impacts was retrieved from two sources, one covering the terrestrial, coastal and freshwater ecosystems (the human footprint, Venter et al., 2016a, 2016b), and the other covering the marine food webs (the cumulative human impact on the world's ocean, Halpern et al., 2015). The human footprint combines the following disturbance drivers: built environments, population density, electric infrastructure, croplands, pasture lands, roads, railways and navigable waterways (Venter et al., 2016a). The cumulative human impact on the world's ocean considers the following impacts on global marine ecosystems: land‐based stressors (nutrient pollution, organic and inorganic pollution, direct human light pollution); fishing‐related stressors (demersal destructive, demersal non‐destructive high and low bycatch, pelagic high and low bycatch, artisanal); climate change stressors (sea surface temperature anomalies, ultraviolet anomalies, ocean acidification) and ocean‐based stressors (sea level rise, commercial shipping, invasive species, ocean‐based pollution, benthic structures) (Halpern et al., 2015).

### Fuzzy categorisation of food webs

Rather than imposing a hard structural topology on food webs, we acknowledge that empirical food webs display a gradient of similarity to a‐priori‐defined network structures. Focusing on degree distribution, that is, the frequency distribution of the number of links that every node in the food web has with other nodes, we devised an approach to characterise each food web based on the distance that its empirical degree distribution topology has to each one of the pure topologies usually considered, scale‐free and random. First, we compared the shape of the degree distribution with both, a power‐law curve (characteristic of scale‐free networks) and a Gaussian curve (characteristic of random networks). There is still some discussion on the functional form of the degree distributions with longer tails, with authors considering either that food webs have power‐law degree distributions (Montoya & Solé, 2002), or that these are closer to an exponential distribution rather than a power‐law (Dunne et al., 2002a; Marina et al., 2018). Here, we wanted to test explicitly how close the food webs were to a scale‐free network, as described by Albert et al. (2000). As such, we used power‐law and Gaussian curve fitting to explore the extent to which the food webs in our database are closer to scale‐free or random degree distributions respectively. Then, each food web was plotted in a plane defined by the fit to each of these curves. Knowing the position of the scale‐free and random topologies in this plane, we measured the Euclidean distance between each food web and these positions to characterise the food web structure.

Scale‐free and random topologies are defined by the homogeneity of the degree distribution, as defined by Solé and Valverde (2004). The degree distribution was considered to be either highly heterogeneous (few nodes highly connected), as with scale‐free networks, or highly homogeneous (the number of connections is nearly equivalent across nodes), as with random networks.

#### Degree distribution

We calculated the degree (*ki*), that is, the number of connections that each node (species) in the food web (*i*) has to other nodes, and computed the histogram of non‐cumulative, non‐binned (the degree frequencies were not grouped into classes), degree distributions, *p*(*k*) (frequency distribution of the number of links per node). The shape of the histograms' curve was then used to assess the extent to which food webs can be categorised into scale‐free and random frequency distributions.

#### Curve fitting and Pearson correlation coefficient (*R*)

We fitted the resulting food web's degree histogram, *p*(*k*), with power‐law $P^{\mathrm{PL}}\left(x\right)$ and gaussian $P^{G}\left(x\right)$ curves. The best‐fitting parameters were calculated applying the Nelder–Mead method (Nelder & Mead, 1965), which performs unconstrained nonlinear minimisation of the sum of squared residuals with respect to its parameters. The correlation coefficient between the sampled dataset *p*_*j*_ and the fitted dataset *P*_*j*_ is defined as (Weisstein, 2021):

(1) $$R=\frac{{\mathrm{SS}}_{\mathrm{reg}}}{{\mathrm{SS}}_{\mathrm{tot}}}$$

where:

$${\mathrm{SS}}_{\mathrm{reg}}=\sum {\left[P_{j}-\left\langle p\right\rangle \right]}^{2}$$

$${\mathrm{SS}}_{\mathrm{tot}}=\sum {\left[p_{j}-\left\langle p\right\rangle \right]}^{2}$$

are the ‘regression sum of squares’ and the ‘total sum of squares’ respectively, with $\left\langle p\right\rangle$ being the average of the measured degree frequencies.

The value of *R* varies within the range $\left[-1,1\right]$, with a value of *R* = 1 implying that the relationship between *P* and *p* can be described by a linear equation (linear correlation); *R* = −1 implying that *P* and *p* are anti‐correlated, and *R* = 0 implying that there is no linear correlation.

#### Power‐Law heuristics

We fitted sampled histogram *p*_*j*_ set points using a generalised Power‐Law function, $P^{\mathrm{PL}}\left(k\right)=a*k^{b}+c$, with parameters *a, b, c* and computed the best‐fitting parameters and *R*_PL_ for each food web in our database.

#### Gaussian heuristics

We fitted sampled histogram *p*_*j*_ set points using a generalised Gaussian function, $P^{G}\left(k\right)=a*e^{-\frac{{\left(k-\mu \right)}^{2}}{2\sigma^{2}}}+b$ with parameters *a, b, μ* (mean), *σ* (standard deviation).

We computed the best‐fitting parameters and *R*_*G*_ for each food web in our database. We finally imposed the following criteria:

(2) $$R_{G}=\left\{\begin{array}{c} R,\text{if}\;\mu +\frac{\sigma }{3}>0\;\text{and}\;k_{\max}>\mu +\sigma \\ 0,\text{otherwise} \end{array}\right.$$

to ensure that only food webs with both parts of the Gaussian bell‐shaped curve of the degree distribution were considered. All others, not conforming with the conditions defined in Equation (2) were not considered (with *R*_G_ being set to zero) (Figure S5).

#### Category membership

Considering we got *R* ∈ [0,1] for all food webs, *R*_G_ and *R*_PL_ can be used as a surrogate to gaussian and power‐law categories of membership probabilities (Figure 1, panel h). By plotting each food web in the (*R*_G_, *R*_PL_) plane and deriving the distance to pure topologies (Figure 2), we assessed the similarity of each food web with the pure topologies. If, for instance, a food web has an *R*_G_ close to 1 and an *R*_PL_ close to 0 then it would be plotted closer to the random pure topology. On the other hand, a food web with an *R*_G_ close to 0 and an *R*_PL_ close to 1 would be plotted closer to the scale‐free pure topology.

As we identified pure categories (scale‐free and random) on the (*R*_G_, *R*_PL_) plane, we characterised the food webs by computing the Euclidean distance to each pure topology: ‘distance to power law’ (D2PL), and ‘distance to pure gaussian’ (D2PG), as shown in Figure 2.

While focusing on a single descriptor of food web structure, that is, degree, which is receiving increasing attention by ecologists (Araújo et al., 2011; Jordano et al., 2003; Poisot & Gravel, 2014), we avoid issues related to the covariance of different food web properties (Vermaat et al., 2009).

### Relating food web structure to human pressure

To explore the relationship between the Euclidean distance of each food web with the pure category in the *R*_PL_/*R*_G_ plane and human pressure, the distances were binned as follows: the number of bins in each graph was chosen by an optimisation procedure by which we chose the maximum number of bins with *R* > 0.8 (as shown, with an example, in Figure S2). Finally, we calculated correlations between distances to pure topologies and human impact, by resorting to linear regression.

### Evaluation of food robustness to species extinction

We evaluated the robustness of individual food webs to species extinctions by simulating species removal. We followed the strategy proposed by Bellingeri et al. (2013), by which the species removal follows a gradient of intentionality (*I*) varying from 0 to 1. If *I = 0* the removal of nodes is random. If *I = 1* the removal of targets preferentially highly connected nodes, that is, hubs. The probability of each node being removed is derived from the family of exponential probability mass functions (Equation 3):

(3) $$P_{E}\left(K|I\right)=\frac{{\left(1-I\right)}^{\left(k_{\max}-k\right)}N_{k}}{\sum_{i=k_{\min}}^{i=k_{\max}}{\left(1-I\right)}^{\left(k_{\max}-i\right)}N_{i}},0\leq I<1$$

where *k* is node degree, *k*_min_ and *k*_max_ are the minimum and maximum number of trophic interactions, *N*_*k*_ is the number of nodes with degree *k* and *N*_*i*_ is number of nodes with degree *i*.

Then we used a metric of network robustness (*R*_50_), as follows (Equation 4):

(4) $$R_{50}=\frac{E}{S}$$

where *E* is the number of primary extinctions required to cause 50% of species to be extinguished and *S* is the total number of nodes. Due to the stochastic nature of these simulations, but considering the computation requirements, we simulated 100 repetitions of each parameter set.

In agreement with previous research (Bellingeri et al., 2013), we expected food webs closer to the scale‐free distribution of degree, to have an R50 shaped like a decreasing sigmoid curve, showing a threshold in the intentionality index, at which the probability of removing hubs is higher. Random food webs, in particular, would have a more linear response of R50 with intentionality.

### Food web structure and response to disturbance

Having characterised each food web according to the distance to each one of the two pure topologies considered, and after evaluating how each one of them would respond to disturbance (in the form of species extinctions), the next logical step was to evaluate if structure was related to the estimated food web robustness in our empirical dataset. To assess such a relationship, we fitted a cumulative Weibull function (describing a sigmoid curve; Equation 5) to each of the robustness plots (Appendix S2).

(5) $$y=a\left(1–\exp\left(-b.x^{c}\right)\right)$$

The shape of the sigmoid curve is determined by parameters *b* and *c*. We retrieved, to each food web, values for the parameters *b* and *c* (in Equation 5).

We expected different topologies to react differently to an increasingly directed attack, depending on degree distribution homogeneity. A food web closer to scale‐free would be robust to attacks until a given level of intentionality, corresponding to the extinction of poorly connected species (which are the most of the species in these networks). As intentionality increases, expressing a higher probability of attacking hubs, we expect a sharp decrease in R50, which should be described by a sigmoid curve. In random food webs, as we move along a homogeneity gradient concerning the degree distribution, we would not see such a threshold in the shape of R50. In these networks, particularly the random where most species has roughly the same number of trophic interactions, the intentionality of the attack does not affect robustness. As such, we expect the R50 to be more linear.

The datasets were retrieved from online databases and pre‐processed using the FWebs R package (Mestre, 2022). The value for the anthropogenic impact on each food web was obtained using the function *extract* from the package raster (Hijmans, 2022) in the R software, version 4.1.2 (R Core Team, 2021). The evaluation of food web robustness to disturbances was conducted in R, with code available in the Figshare repository. Data regression and plotting were conducted using Matlab (MATLAB, 2010).

## RESULTS

The food webs examined were predominantly closer to the random topology (average distance to pure scale‐free: 0.870; average distance to pure random: 0.773).

**FIGURE 3:**
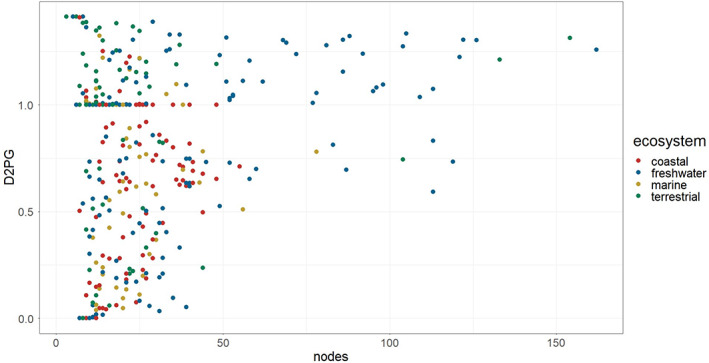
Plot depicting the relation between distance to pure gaussian (D2PG) and the number of nodes in each food web (nodes). Notice that distances to pure scale‐free (D2SF) are symmetrical with D2PG so that high values of D2PG corresponded to low values of D2SF and vice versa.

Several food webs in our dataset are small. Although small size can limit the capacity to classify food webs according to topological profiles, removing small food webs would truncate a pattern of interest: larger food webs tend to be located in regions of lower human disturbance, while smaller food webs are present almost exclusively in regions with greater human disturbances (Figure S4). Removing small food webs would thus bias sampling towards food webs generally exposed to lower impacts. Also, it would limit the capacity to discern clear topological patterns. Smaller food webs span through a variety of topologies, from scale‐free to random, if anything favouring random ones (Figure 3), while bigger food webs tend to be closer to scale‐free (D2SF is symmetrical to D2PG, hence with high values in the y axis represent low D2SF) (Figure 3; Figure S4).

**FIGURE 4:**
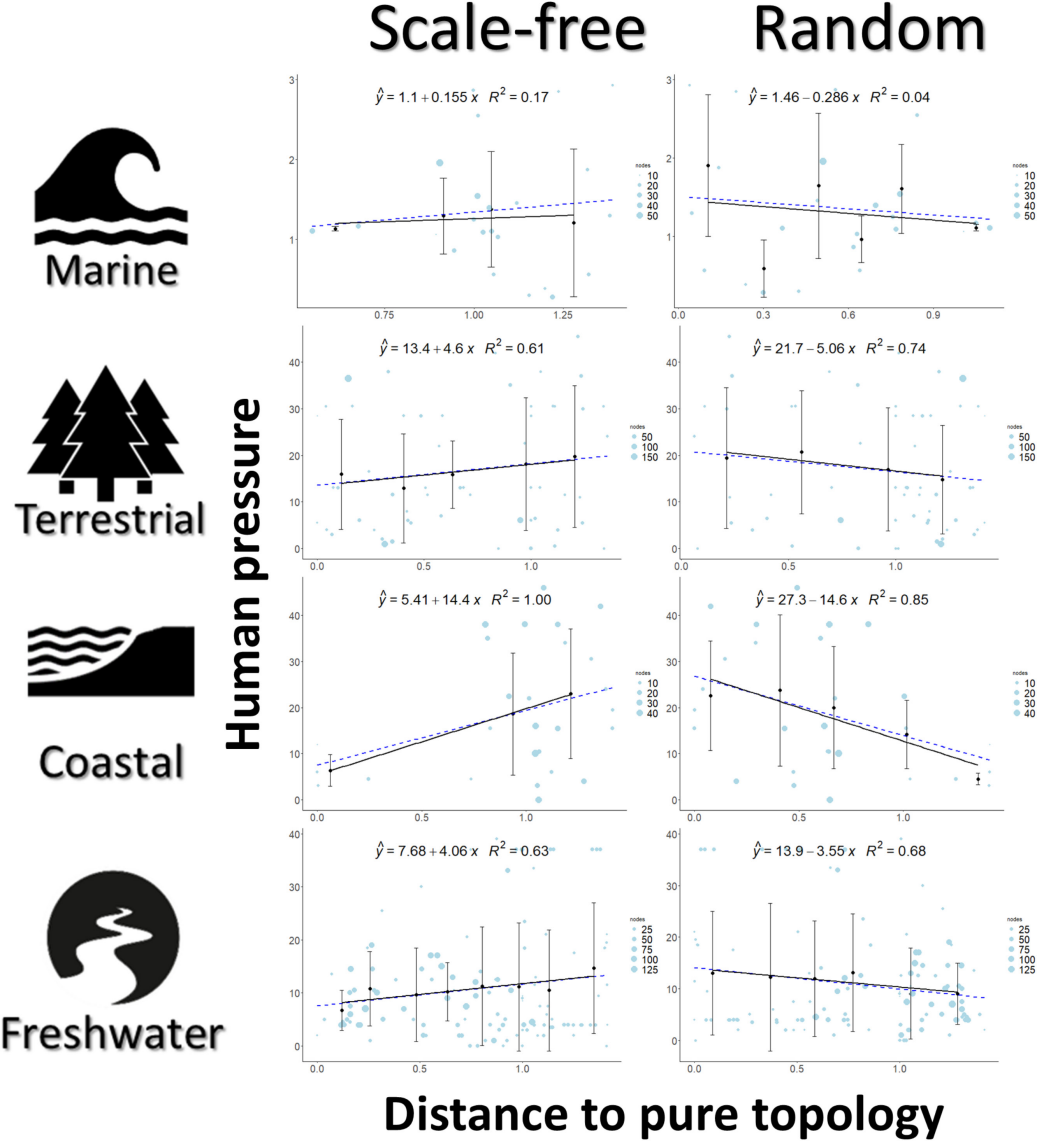
Relationship between human pressure and the distance to each of the three pure topologies in each ecosystem. Human pressure was evaluated as the cumulative impact on the world's oceans (Halpern et al., 2015) on coastal and marine ecosystems and the human footprint (Venter et al., 2016a, 2016b) impact on terrestrial and freshwater ecosystems. The relation between distance to pure topologies and the human pressure was characterised by the best fitting linear relationship. Note that values in the x‐axis represent the distance to pure topologies (D2PG and D2PL) to each food web (as shown in Figure 2). As such, values greater than 1 are possible, only the RG and RPL are constrained to vary between 0 and 1. Blue circles represent the raw data (scaled by food web size) and the blue dashed line represents the regression on these data. The black points and the black regression line represent the binned data. Error bars express the variance of the averaged values in each bin. The regression equation represented refers to the regression on the binned data.

Consistent with predictions, we found that distance to pure scale‐free topology is generally positively associated with increasing human disturbance (Figure 4). That is, food webs closer to the scale‐free are mainly found in less impacted regions. In contrast, food webs with a topology closer to random are generally negatively associated with increasing levels of disturbance and found mainly in regions with high human footprint (Figure 4). Note that despite binning to help visualise noisy data (see methods), the slope of the regression on the raw dataset (the blue dashed lines in Figure 4) is similar to the regression using binned values (black lines in Figure 4). Only for marine food webs, our prediction is not verified with distance to scale‐free responding negatively to increased human disturbance.

**FIGURE 5:**
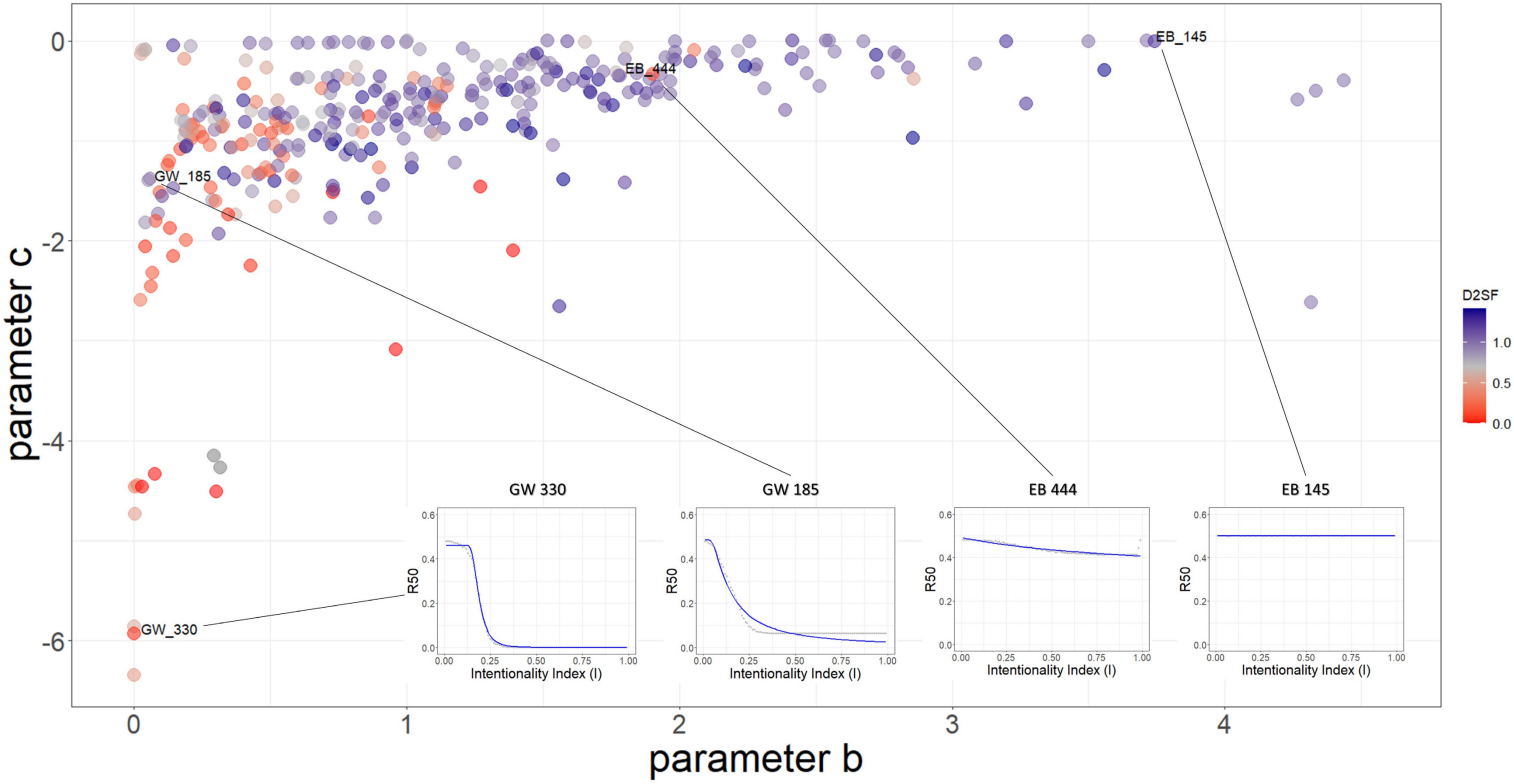
Location of the food webs in the parameter space defined by the parameters (b) and (c) in Equation 5. The gradient, from red to blue, refers to the increasing distance to pure scale‐free). The parameters (b) and (c) in Equation 5 determine the shape of the R50 curve. The smaller plots (those in food webs GW330, GW185, EB444 and EB145) depict the robustness (R50) curve as the intentionality increases (the R50 plots for all the food webs are available as Appendix S2). Within each R50 plot: The grey points represent the average of R50 with increasing intentionality. The blue line refers to the cumulative Weibull function used to fit the R50 values.

Consistently with simulations of random and targeted attacks on virtually constructed networks (Albert et al., 2000), our simulations on empirical food webs show that robustness to node removal is linked to degree distribution (Figure 5). Food webs with degree distribution closer to scale‐free (predominant in less impacted regions) tend to show a sigmoid curve in the response to increasingly intentional disturbance (increasing probability of extinguishing hubs): the R50 (Equation 4) is relatively stable until it decreases abruptly (Figure 5). This threshold represents the point at which hubs have a higher probability of being targeted by removal. On the other hand, as node homogeneity increases (as with random food webs) robustness to an increasingly intentional species removal becomes more linear (Figure 5, and Appendix S2 of the Supporting Information).

## DISCUSSION

Whether food web topologies are predictable from external factors, such as environmental and/or human‐induced stressors, is a longstanding question in ecology (Pimm, 1980; Pimm et al., 1991; Pimm & Ktiching, 1987). Previous theoretical simulations suggest that networks, not just food webs, are differentially affected by random or targeted attacks (Albert et al., 2000). The idea of ‘attacks’ on networks is relatively abstract but, in ecology, it can be translated into disturbance, that is ‘an event or force, of nonbiological or biological origin, that brings about mortality to organisms and changes in their spatial patterning in the ecosystems they inhabit’ (Paine, 2015). Disturbances in ecosystems can be random or target specific components of the ecosystem. The former originates in the absence of extraordinary events, including background processes, such as environmental stochasticity or ecological drift causing species local abundances, extinctions and colonisations to fluctuate around a central tendency (Lande, 1993; Quental & Marshall, 2013). The latter can be assimilated to extraordinary non‐random events affecting the persistence or movement of specifically sensitive traits, populations or communities. Global to regional extinction events, following climate changes or other human‐induced disturbances are examples falling in this category given that they were selective to the traits targeted (bad genes) as well as locations (bad luck) (Hof et al., 2010; McKinney, 1997; Purvis et al., 2000; Raup & Gould, 1993; Thuiller et al., 2011).

Using a spatially distributed empirical dataset of food webs, we tested the prediction that different levels of disturbance would affect food web topology differently. Despite noise in the data, we found that the predicted trajectories are generally met. Regions subjected to higher levels of human disturbances (that we propose should lead often to a structure of targeted attacks on species within communities) have a predominance of random networks, whereas regions with lower disturbances (that we propose should often coexist with randomness resulting from stochastic dynamic processes) have a greater predominance of scale‐free (with the notable exception of marine and coastal food webs).

Only in the marine food webs did we observe a pattern departing from expectation: a negative relationship between distance to scale‐free and human‐induced disturbance. In other words, in more disturbed regions we found that scale‐free food webs were more common. Consistently, the distance to pure gaussian increased with human disturbance. Marine food webs were found to be fundamentally different from their terrestrial and freshwater counterparts in previous research (Link, 2002): are more connected and have higher omnivory than terrestrial and freshwater food webs. These properties might explain why marine food webs are more resilient and further from scale‐free (Figure 3; Figure S3).

The departure from expected marine food web topology relationships with disturbance needs to be further investigated, as should the generality of the consistency of our observations in terrestrial, freshwater and coastal ecosystems. But another important question arising from our observations concerns the nature of the mechanisms underpinning the adjustment of food web topologies to disturbance intensity. A parsimonious mechanistic explanation is that the different structures of ‘attacks’ (random versus targeted, here postulated to covary with the intensity of human disturbances) generate different network topologies by node deletion (local extinction). With time, should the structure of the attacks be persistent, topologies with greater levels of resilience to specific types of attacks would be favourably selected (Devictor et al., 2008; de Visser et al., 2011; Kitahara & Fujii, 1994; Start et al., 2020). An example of this are intermittent rivers in arid regions, which are cyclically affected by drought, destroying the structure of freshwater food webs periodically, but quickly recovering during the wet winter and/or spring (López‐Rodríguez et al., 2012; Power et al., 2013).

The measured association of food web size with disturbance also deserves further investigation. However, it is consistent with previous research demonstrating that food chain length, the number of nodes and links tend to decrease with disturbance (Jenkins et al., 1992; Parker & Huryn, 2006; McHugh et al., 2010; Thompson & Mcintosh, 1998).

If our results were general across scales and systems, we would predict that disturbance‐prone ecosystems would likely favour food webs with a more homogeneous, or random topology of degree (Aspin et al., 2019; Ledger et al., 2011; Peralta‐Maraver et al., 2020). Indeed, disturbance has been shown to promote generalist species in freshwater ecosystems (Canning et al., 2018; Larson et al., 2018). In terrestrial ecosystems, the same tendency has been observed, with diet breadth being a main predictor of susceptibility to habitat fragmentation in vertebrate species (Keinath et al., 2017; Swihart et al., 2003).

Conceptually, the more non‐homogeneous a network is, the higher its dependence on fewer nodes; eliminating these few nodes has a high cost for its stability (Albert et al., 2000). Targeted attacks, such as those predicted within regions with higher levels of human‐induced disturbance, have potentially more severe effects in scale‐free networks than random. Furthermore, the effects of random disturbances are potentially greater in random than in scale‐free networks, considering that the average degree among nodes is higher in the former (Albert et al., 2000).

We further tested this inference with a simulation experiment on the empirical food webs used. We removed species, increasing the probability of extinguishing hubs (the intentionality), and evaluating the impact on the network structure. The resistance to extinctions was evaluated with a robustness index, accounting for the number of primary extinctions required to extinguish 50% of the species in the network. Consistent with initial expectations, scale‐free food webs are resilient to node deletion up until a threshold after which the topology collapses into a different state, while those with topologies closer to random show a more linearised response considering they are not as heavily dependent on hubs.

Several studies have addressed the consequences of species removal on network topology, evaluating the effects of an increasing probability of removing highly interconnected species or hubs, largely supporting the view that, all other things being equal, targeting the removal of highly interconnected species disproportionately increases the number of secondary extinctions (Bellingeri et al., 2013; Dunne et al., 2002b; Eklöf & Ebenman, 2006; Quince et al., 2005). Not all things are equal, however, and the effects of species removal are also differentiated across species with different trophic levels. Generally, the lower the trophic level, the higher the number of secondary extinctions expected (Eklöf & Ebenman, 2006; Staniczenko et al., 2010). Additionally, as Dunne and Williams (2009) alert, the least connected species are not irrelevant, as they can play an important role in the structural integrity of food webs. These authors resorted to simulated food webs and evaluated the robustness to the effects of three types of species extinction, random, prioritising least connected species and more connected species. They concluded that the primary extinction of least connected species might have a substantial impact on the number of secondary extinctions, particularly for food webs with lower connectance. In some cases, the response to the extinction of least connected species is comparable to that of most connected species, revealing the relevance of poorly connected species to maintaining food web structure.

As with other studies using data from several sources, individually recorded for different purposes, there are limitations that need to be recognised. First, the food webs we used were characterised at a variety of scales, from local to oceanic/continental, but the disturbances were derived from maps using interpolations at a particular resolution (Montoya & Galiana, 2017; Raffaelli, 2005; Raffaelli & Moller, 1999). Such mismatches in the scale of the observations can cause errors of association either because the average disturbance values at a given cell might not provide an accurate indication of the disturbance experienced by the food web at a local level (if the food web scale is smaller than the resolution of the disturbance data) or because the area from which the food web was derived encompasses multiple cells, of which the point from which we obtained the disturbance value (the centroid) is not representative (if the food web scale is larger than the resolution of the disturbance data). Second, the same mismatching problems may also arise from measurements occurring in different times (Raffaelli, 2005; Raffaelli & Moller, 1999). No guarantee exists of perfect matching between the timing of the food web observations and the measurements of disturbances, although a great effort was made to reduce this potential source of noise (see methods). Third, food webs are gathered using data collected with different approaches, assembled at different resolutions and examined with different analytical methods (Dunne, 2005; Martinez et al., 1999; Wood et al., 2015). The outcome of such diversity is that food webs may not be fully always comparable (Mestre et al., 2022). Additionally, we used composite indices of human impact. The measurements included have varying effects on food webs and weights given to individual indicators may not reflect their biological impacts appropriately. Lastly, despite the global distribution of our dataset, there are clear spatial sampling biases towards Europe and North America (see also Poisot et al., 2021). Several of the most well‐sampled regions are also the most impacted, leaving underrepresented regions with lower impacts (like tropical areas across South America, South East Asia and Africa). Our dataset also misses important biodiversity areas exposed to high impacts, such as the Indian subcontinent and East Asia.

Other studies examining coarse distributional patterns among properties of trophic structures also found regularities in network topologies. Examining the global structure of marine fish food webs Albouy et al. (2019) found that a low degree of spatial modularity was related to sea surface temperature. A study on vertebrate food webs across Europe (Braga et al., 2019), found that food web metrics (including connectance and mean trophic level) had a non‐random spatial distribution across Europe. In higher latitudes and mountain ranges food webs had fewer species, shorter food chain lengths and a higher proportion of basal species. In central and eastern Europe food webs had higher food chain length and generality (diet breadth). Finally, in southern Europe, food webs were found to be more species‐rich, and have higher link density and clustering coefficient. Yet previous studies, examining food webs described with locally sampled data, have broadly failed to depict general relationships between network topologies and environmental or human‐related variables (Cohen et al., 1993; Dunne, 2005; Pimm et al., 1988). Several shortcomings might have hindered the detection of such relationships but unequivocal generalities between food web topologies and disturbance variables will require confirmation using fully replicated studies across geographical and environmental gradients (e.g. Matias et al., 2017); an endeavour that is becoming possible by coupling metabarcoding with classic survey methodologies (Pereira et al., 2021).

If inferences from simulations, supported by coarse empirical analysis, are confirmed with experimentally derived data, then it would open new perspectives for understanding how ecosystems self‐organise in response to environmental and human‐mediated stressors. Such understanding is critical to predict the long‐term consequences of environmental changes on biodiversity and ecosystem functioning.

## AUTHOR CONTRIBUTIONS

Conceptualisation: MBA, FM, AR; Formal analysis: FM, AR, MBA; Writing: FM, MBA, AR.

### PEER REVIEW

The peer review history for this article is available at https://publons.com/publon/10.1111/ele.14107.

### DATA AVAILAIBILITY STATEMENT

The dataset and code used for the analysis described here were made available in Figshare (doi: 10.6084/m9.figshare.14518362.v3).

## Supporting information


Appendix S1
Appendix S2

Appendix S3
