# Supplementary material for: Human disturbances affect the topology of food webs
Source: Ecol Lett. 2022 Sep 27;25(11):2476–88. doi: 10.1111/ele.14107 (PMC9828725; doi:10.1111/ele.14107)

**Supporting Information for**

**Human disturbances affect the topology of food webs**

**Frederico Mestre, Alejandro Rozenfeld, Miguel B. Araújo**

**Miguel B. Araújo**

**Email: maraujo@mncn.csic.es**

**Appendix 1 – Additional figures**

**Appendix 2 - Robustness plots**

**Appendix 3 (Excel file) – Food web description**

**Appendix 1 – Additional figures**


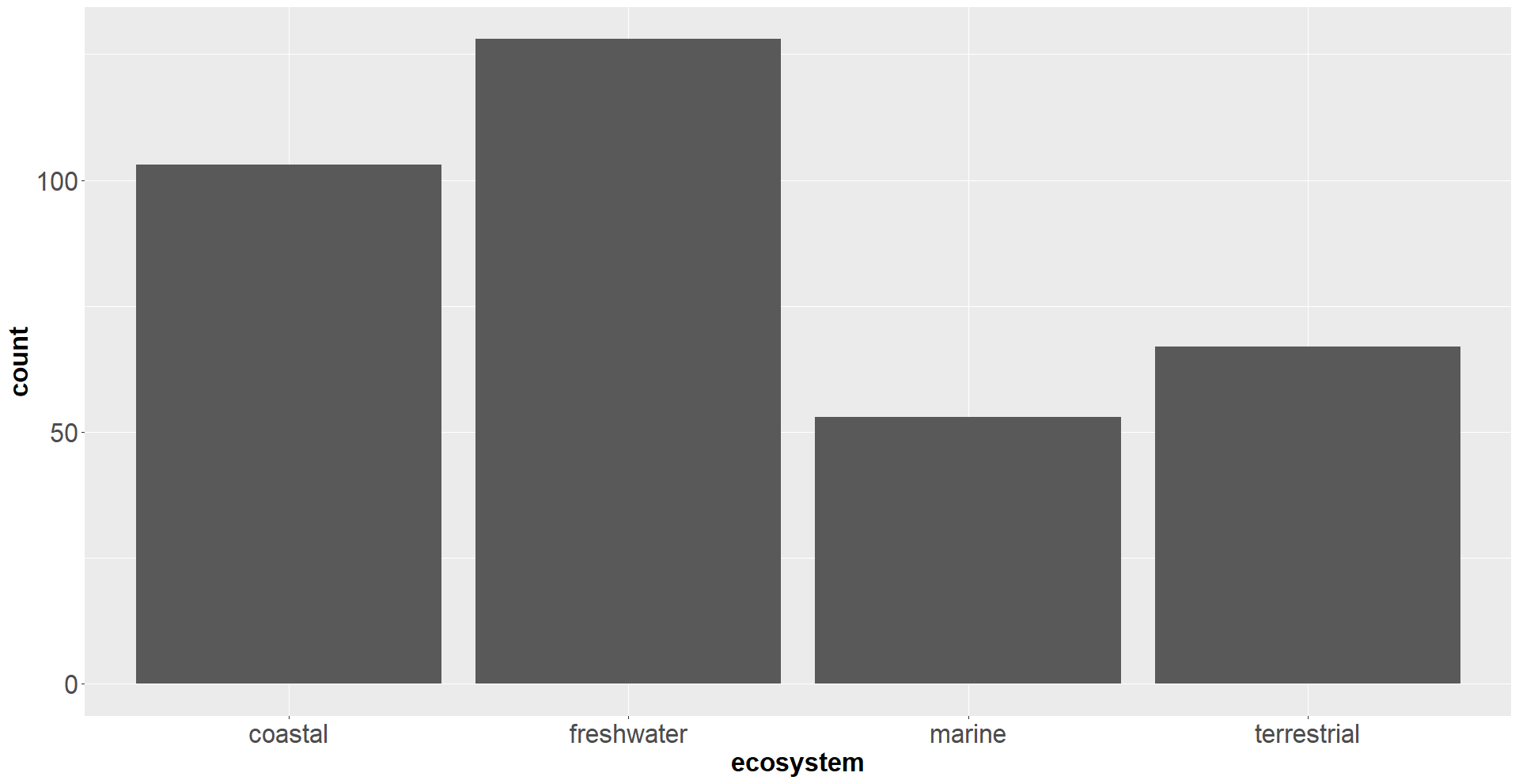


**Fig. S1**. Number of food webs in each of the ecosystem classes considered.


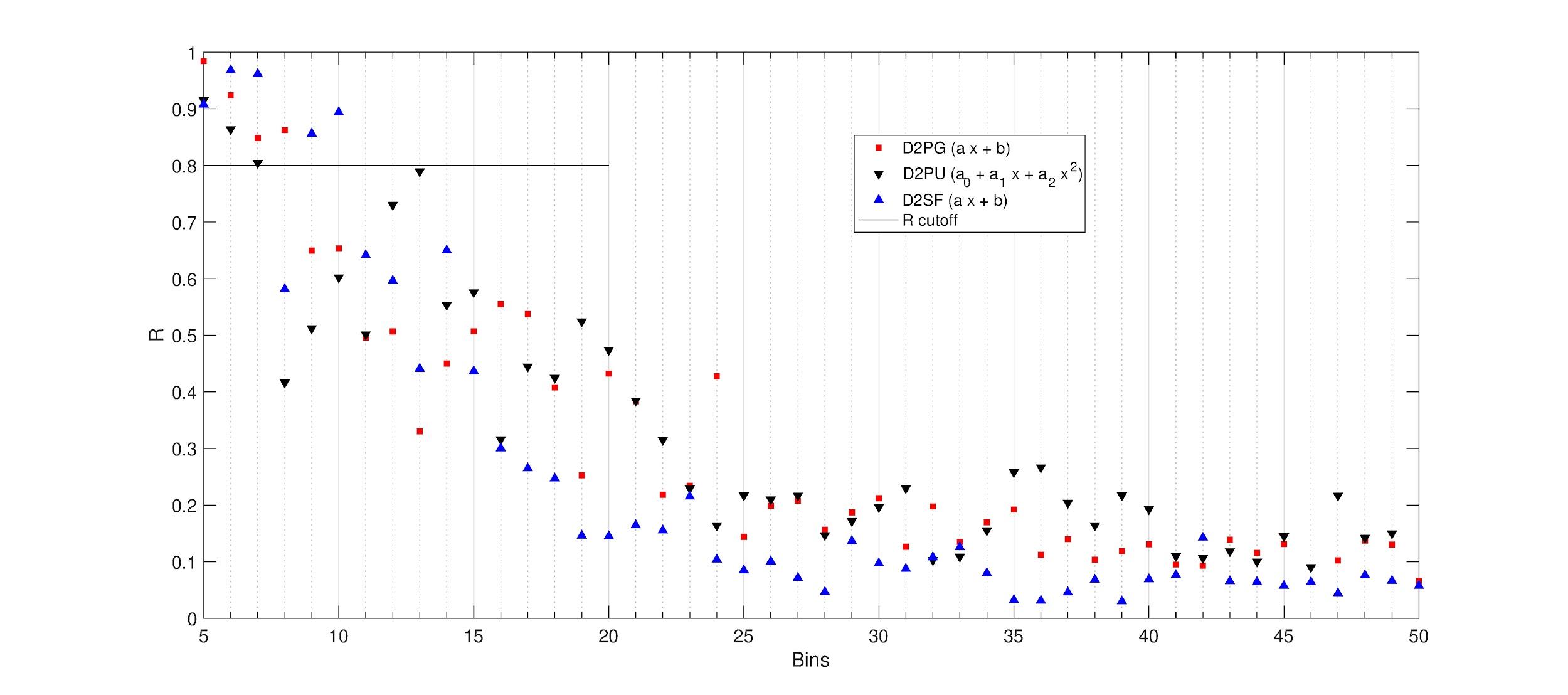


**Fig. S2**. Example plot of the optimization process used to choose the number of bins grouping the distance to each pure topology. Regression coefficient (R) versus the number of bins (bins). The number of bins is the maximum number producing an R > 0.8 (horizontal line). D2PG – Distance to pure Gaussian; D2PU – Distance to pure Uniform; D2SF – Distance to pure Scale Free. In this example, for D2PG (red squares) we would choose 8 bins (the higher number of bins with R>0.8); for D2PU (inverted black triangles) we would choose 7 bins and for D2SF (blue triangles) we would choose 10 bins.


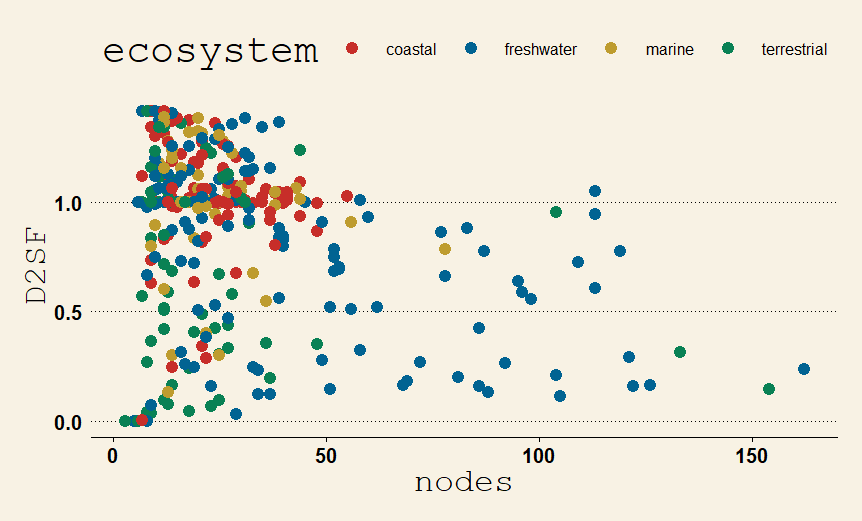


**Fig. S3**. Plot depicting the relation between distance to pure scale free (D2SF) and the number of nodes in each food web (nodes).


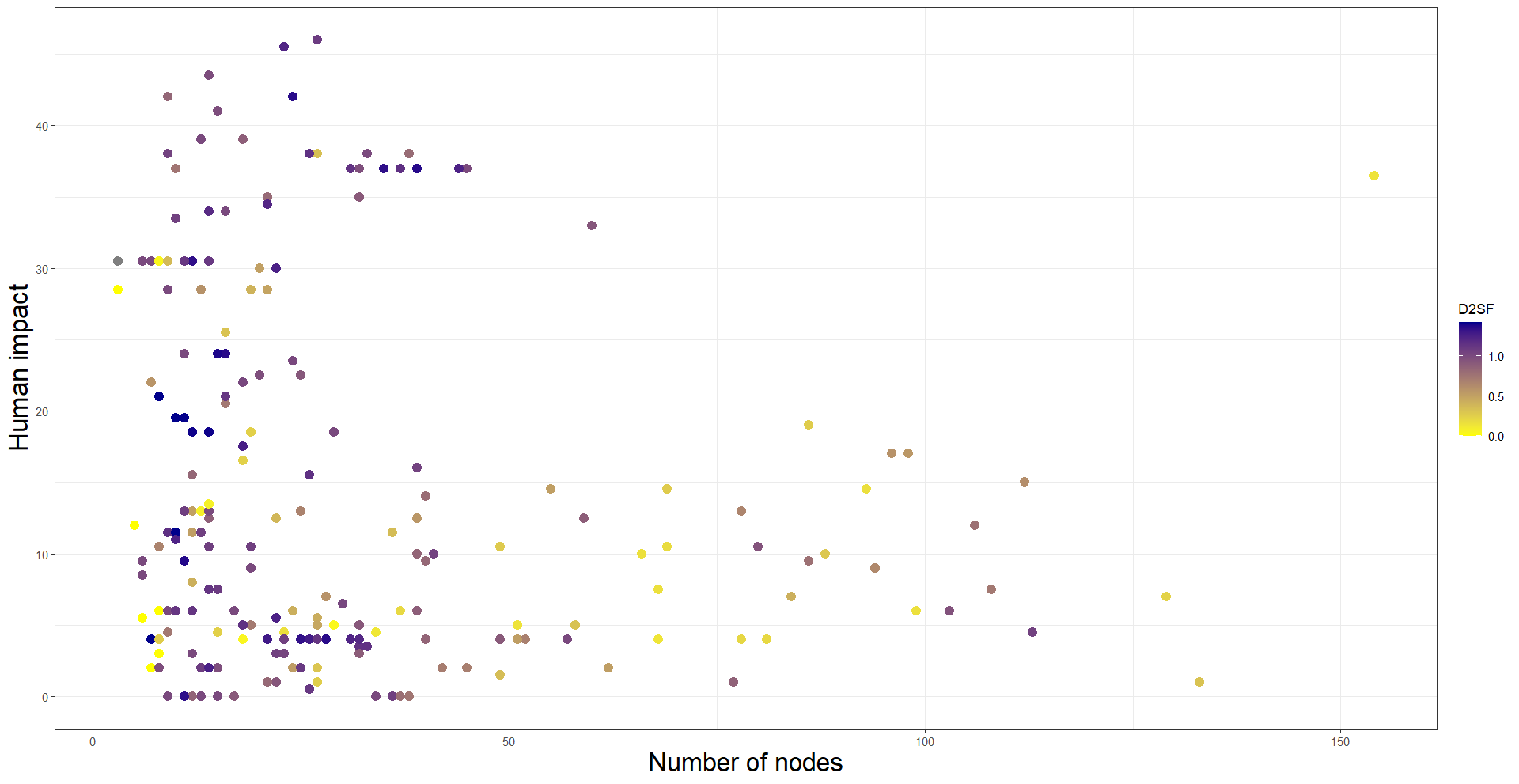


**Fig. S4**. Plot depicting the relation between the number of nodes in each food web and the human impact. Distance to pure scale-free (D2SF): dark blue (closer to scale-free) to light blue (further from scale-free). Bigger food webs, which are mostly closer to scale-free are in regions less impacted by human disturbance.


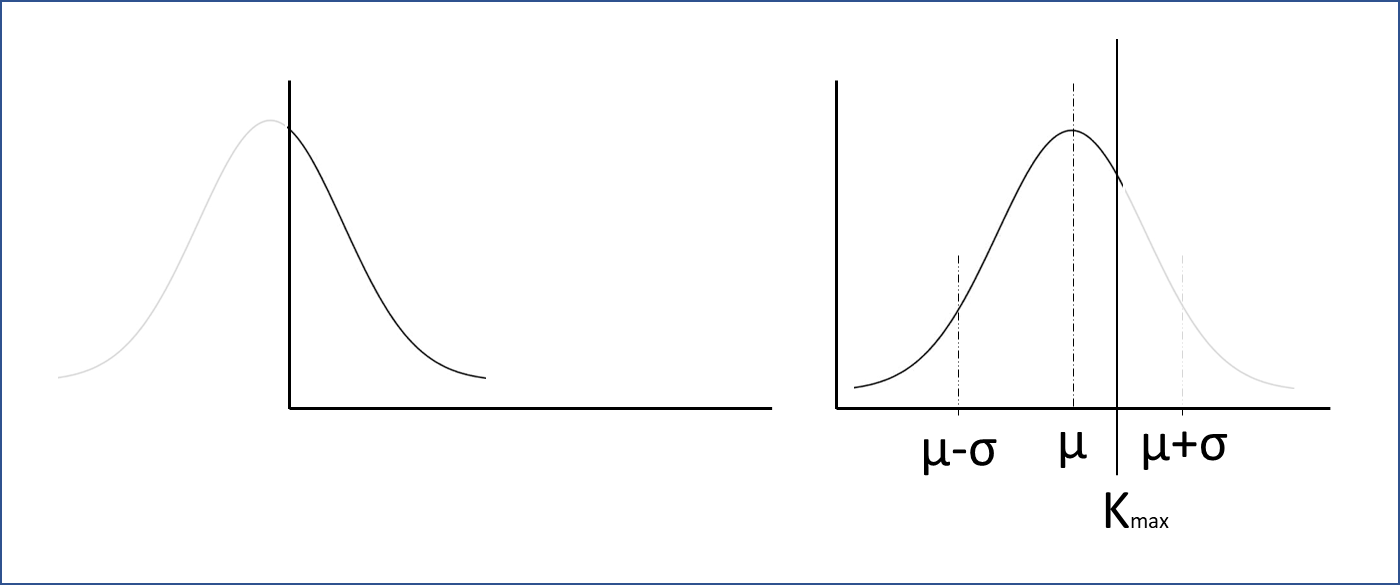


**Fig. S5**. Plots depicting the conditions in which R_G_ was set to zero (eq. 2). Both the left side (*μ + σ/3 > 0*, left plot) and the right side of the curve (*k_max_ > μ + σ*, right plot) must be present for the value of RG to be derived.

**Appendix 2 -** **Robustness plots to each food web**

The following plots depict the Robustness (R50: the number of primary extinctions required to extinguish 50% of the species in the food web as secondary extinctions) across an Intentionality gradient (the intentionality reflects the probability of removing a hub as a primary extinction: I=0 refers to random extinctions and I=1 refers to extinguishing preferentially hubs). The grey points represent the value of the R50 index across the intentionality gradient, the blue line represents the fitted cumulative Weibull function (in some cases it was not possible to adjust a cumulative Weibull – those with a flat R50 - in which case no blue line is depicted).

Food webs showing a marked decrease in R50 (an inverted sigmoid curve) are closer to scale-free, as shown in figure 5 of the main text. The threshold is caused by the extinction of hubs, which will cause a high proportion of secondary extinctions. As such, if hubs are attacked directly (increased intentionality), fewer primary extinctions are required to extinguish 50% of the species in the food web, thus abruptly lowering the R50.


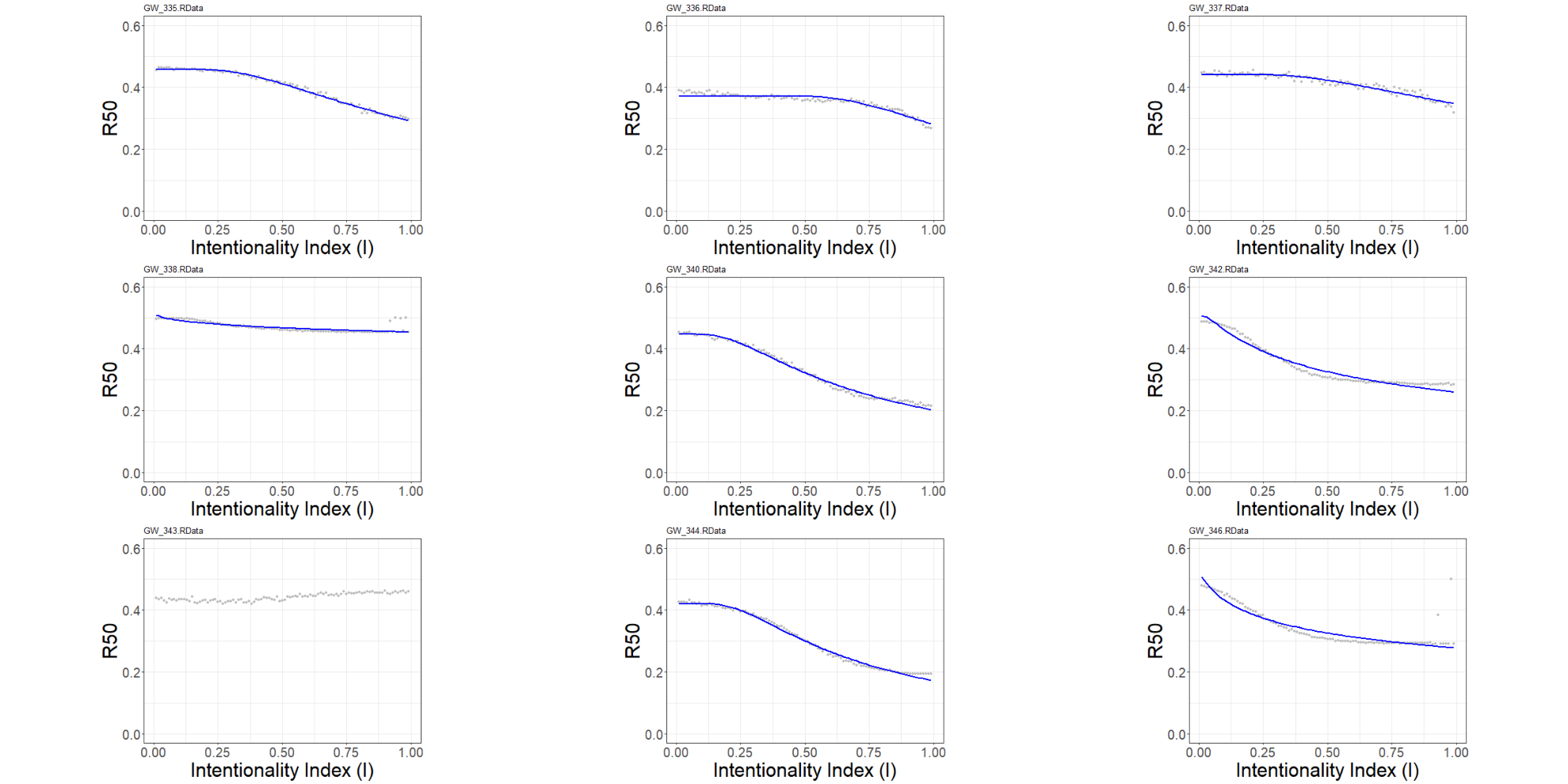

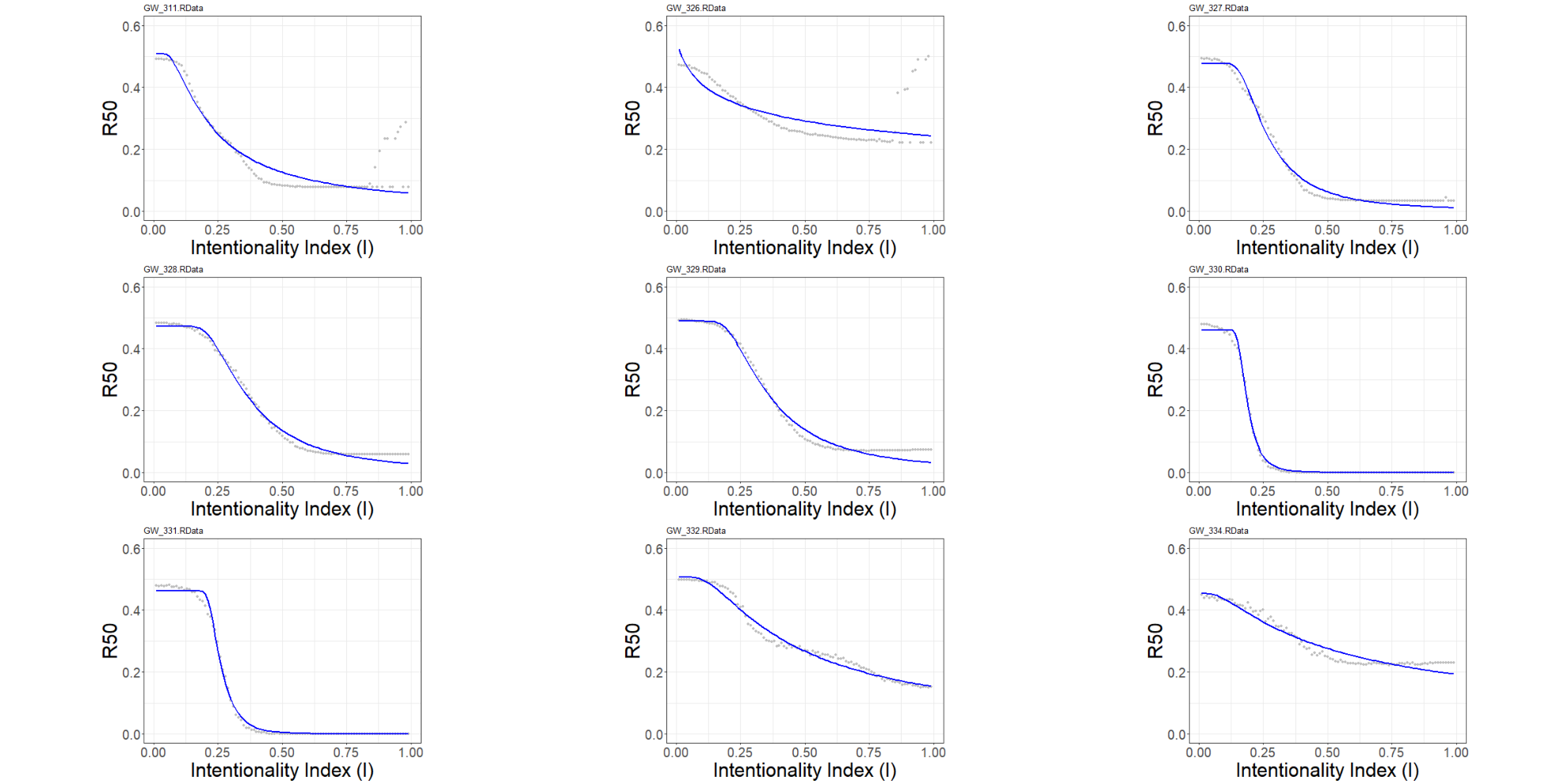

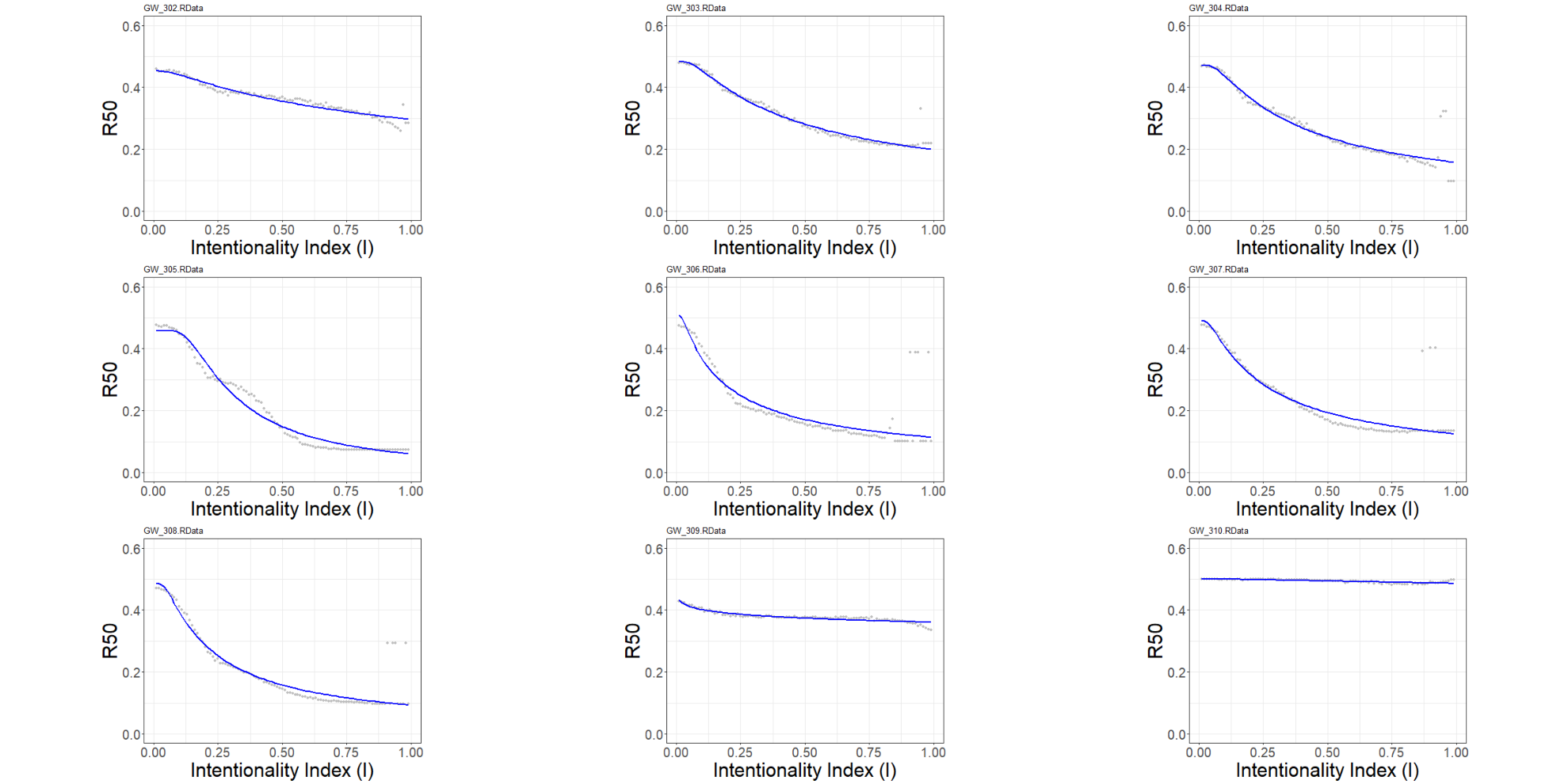

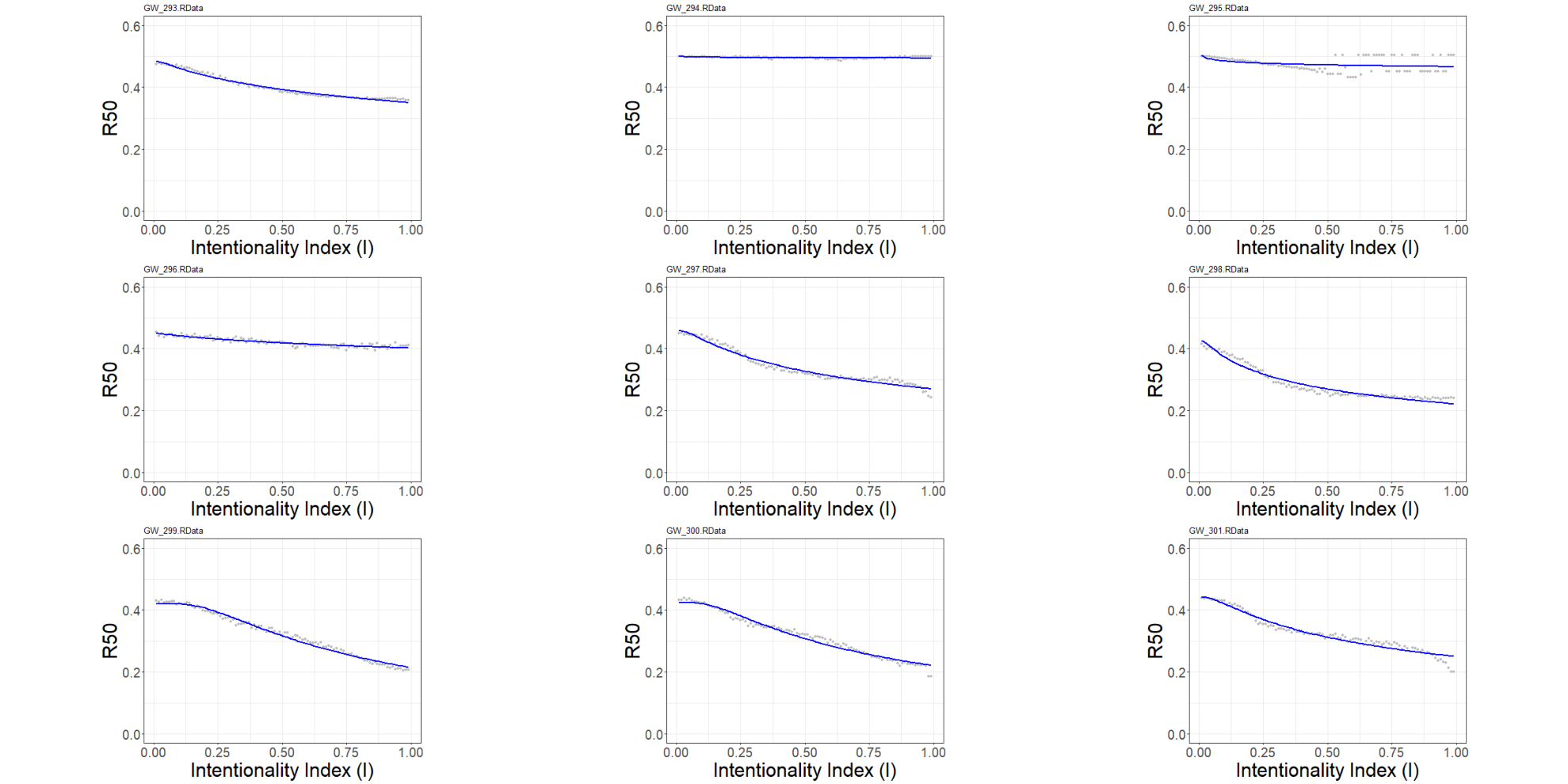

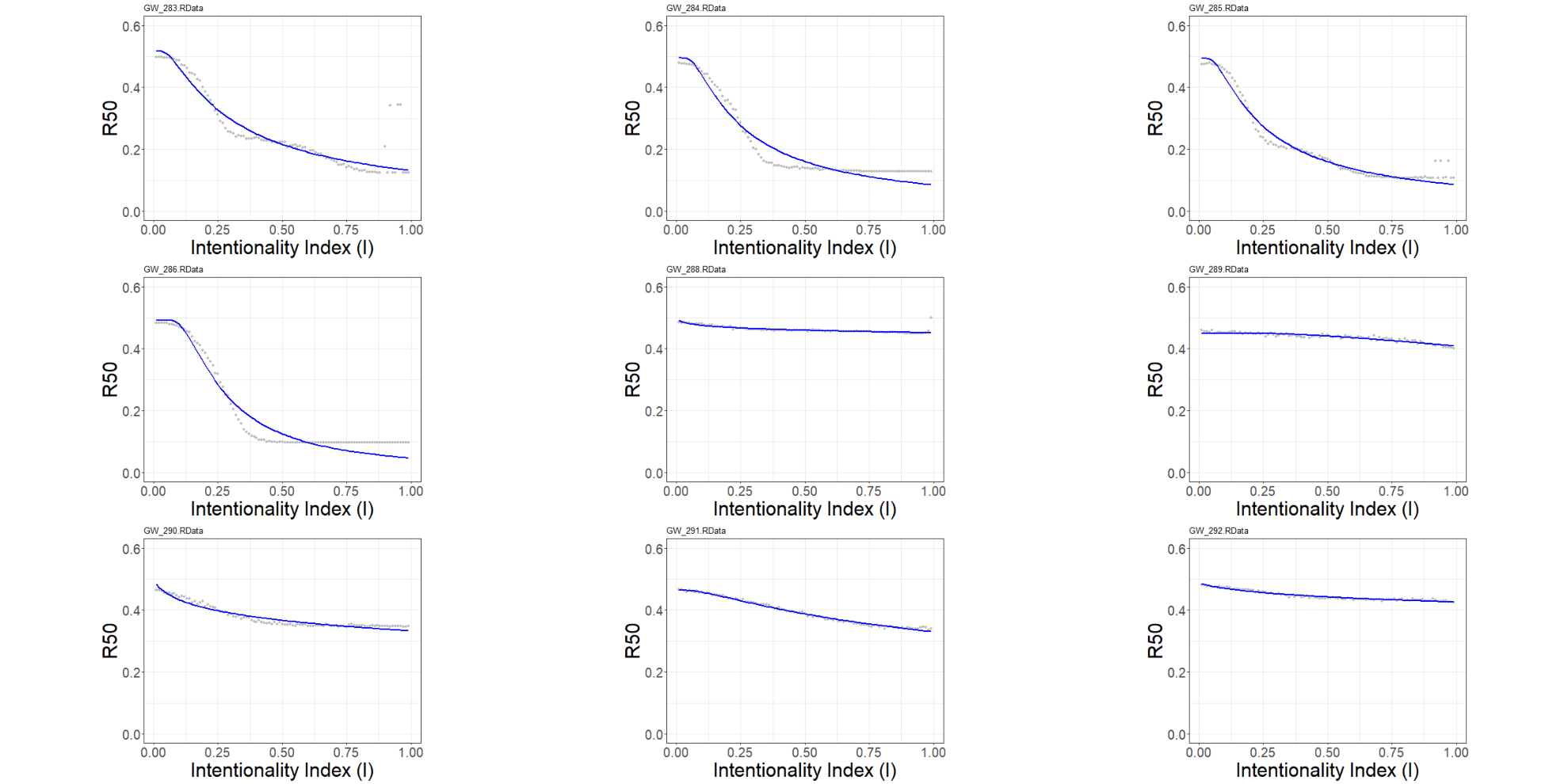

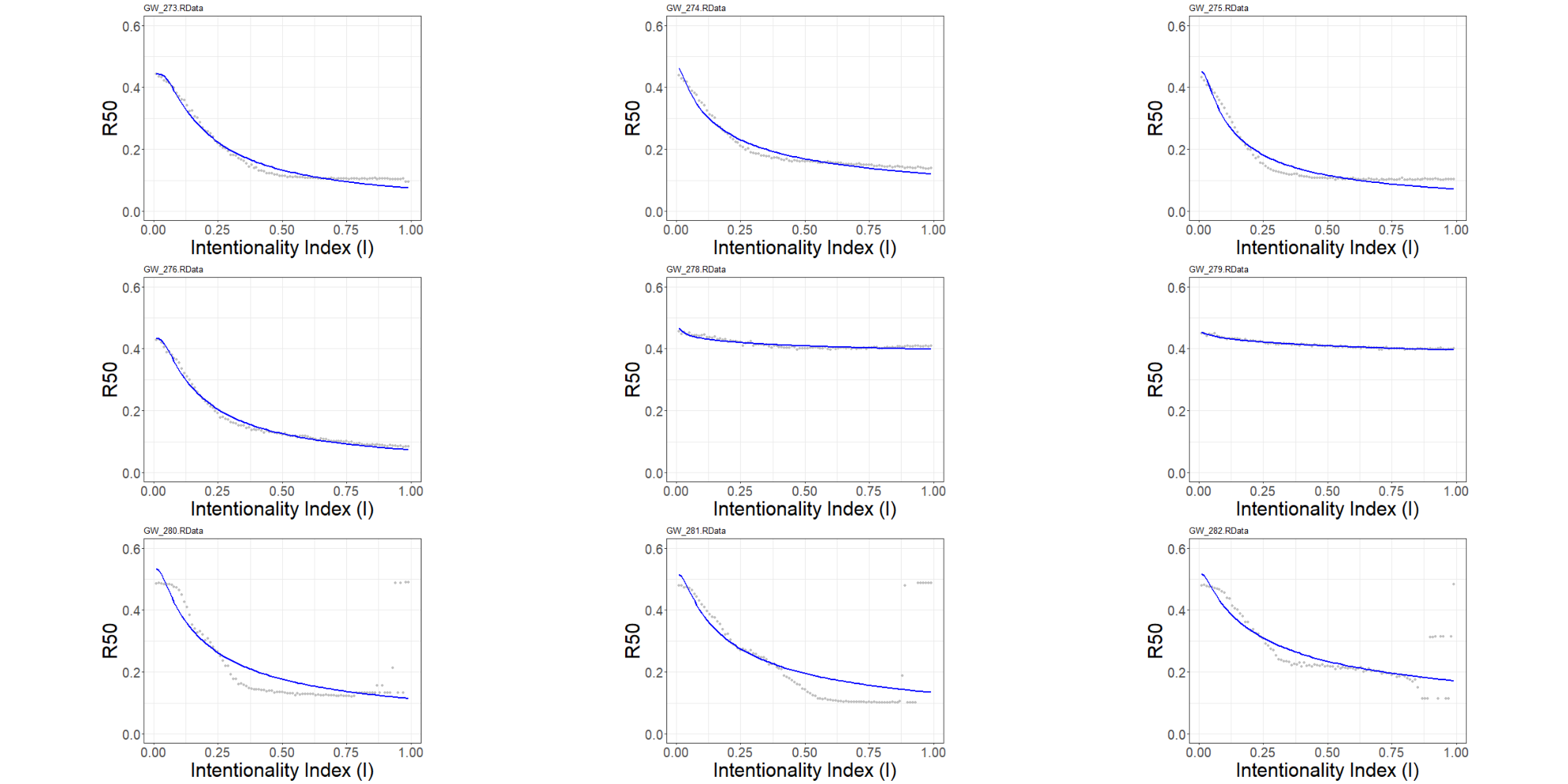

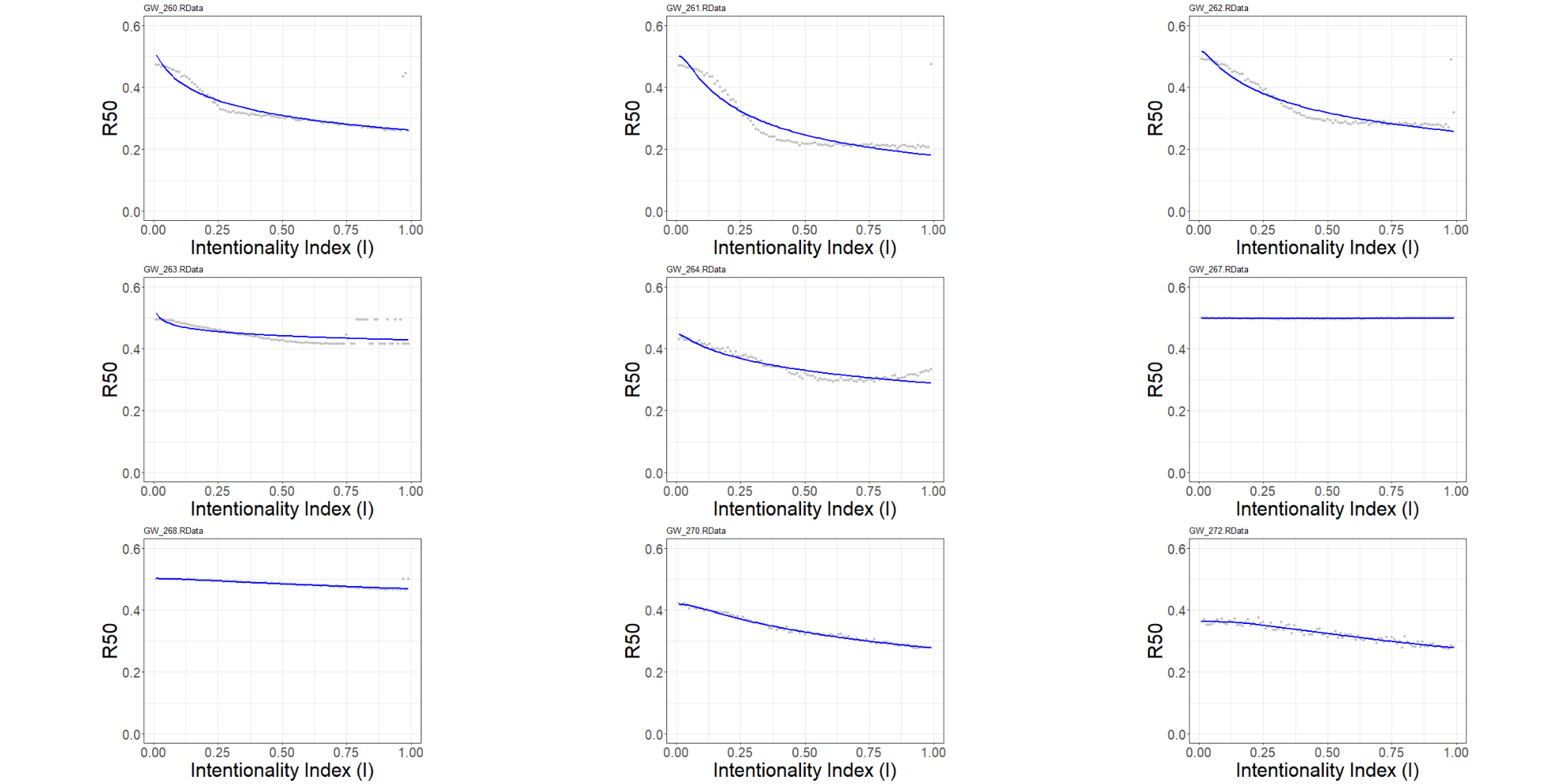

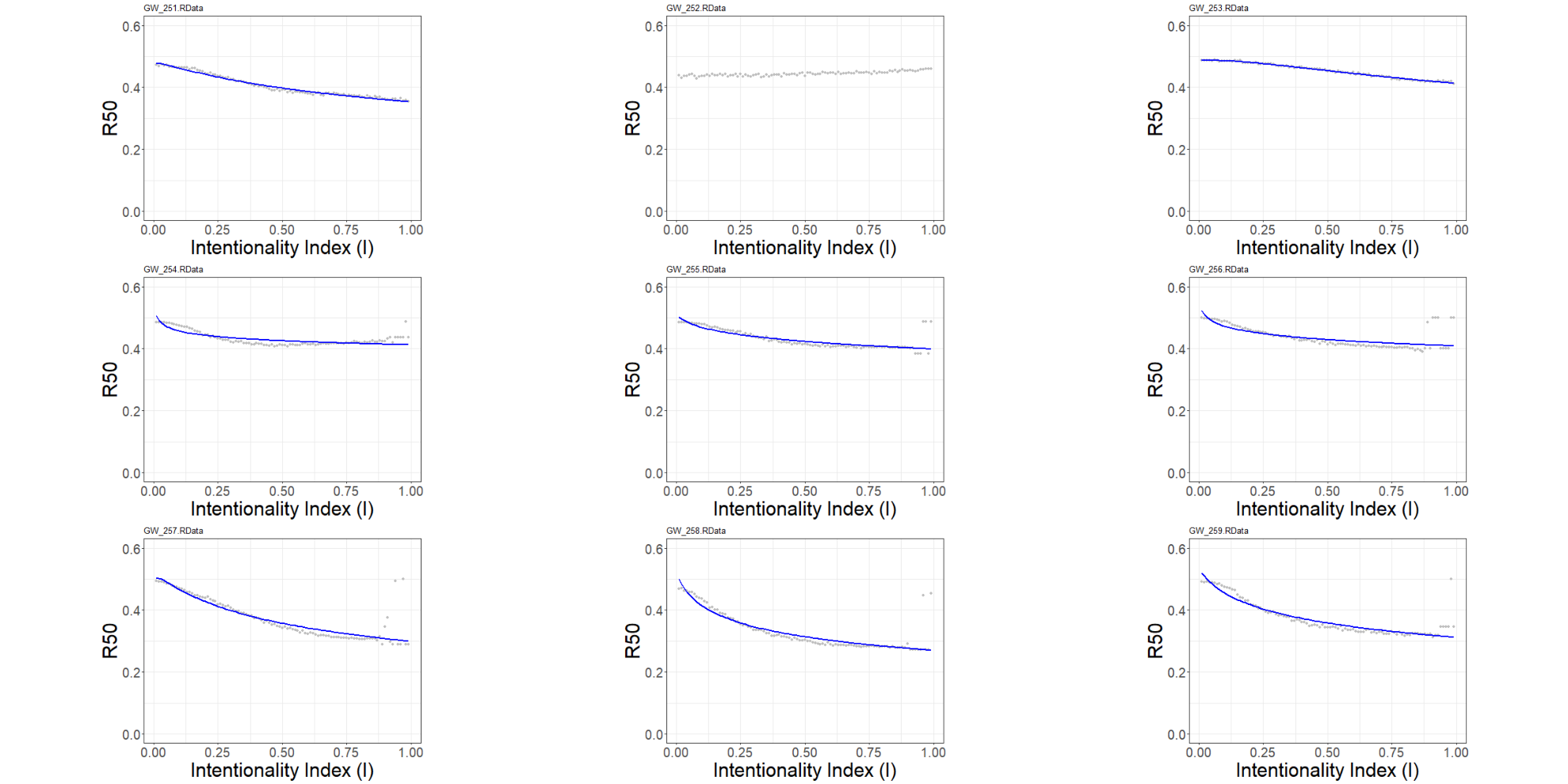

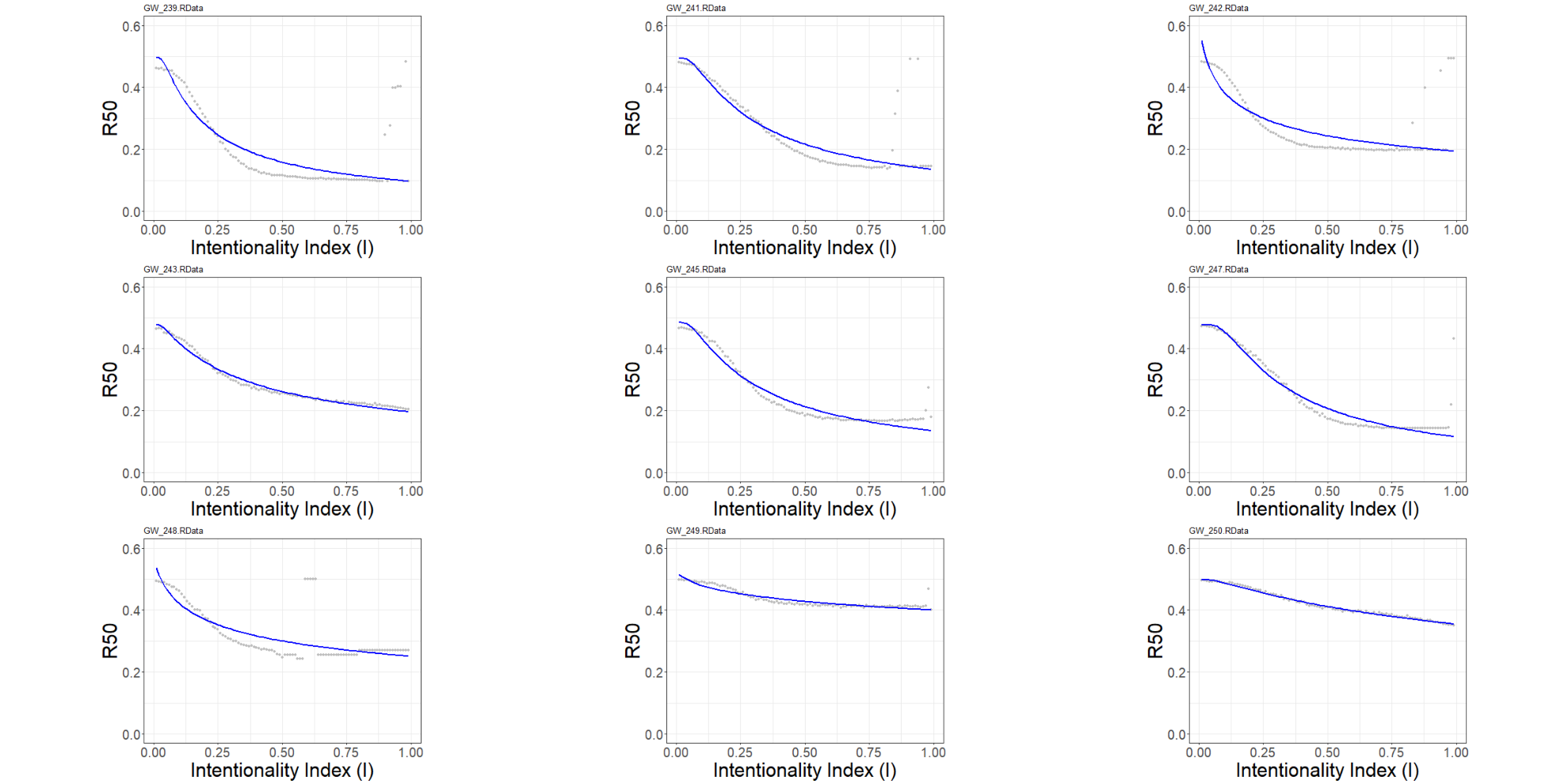

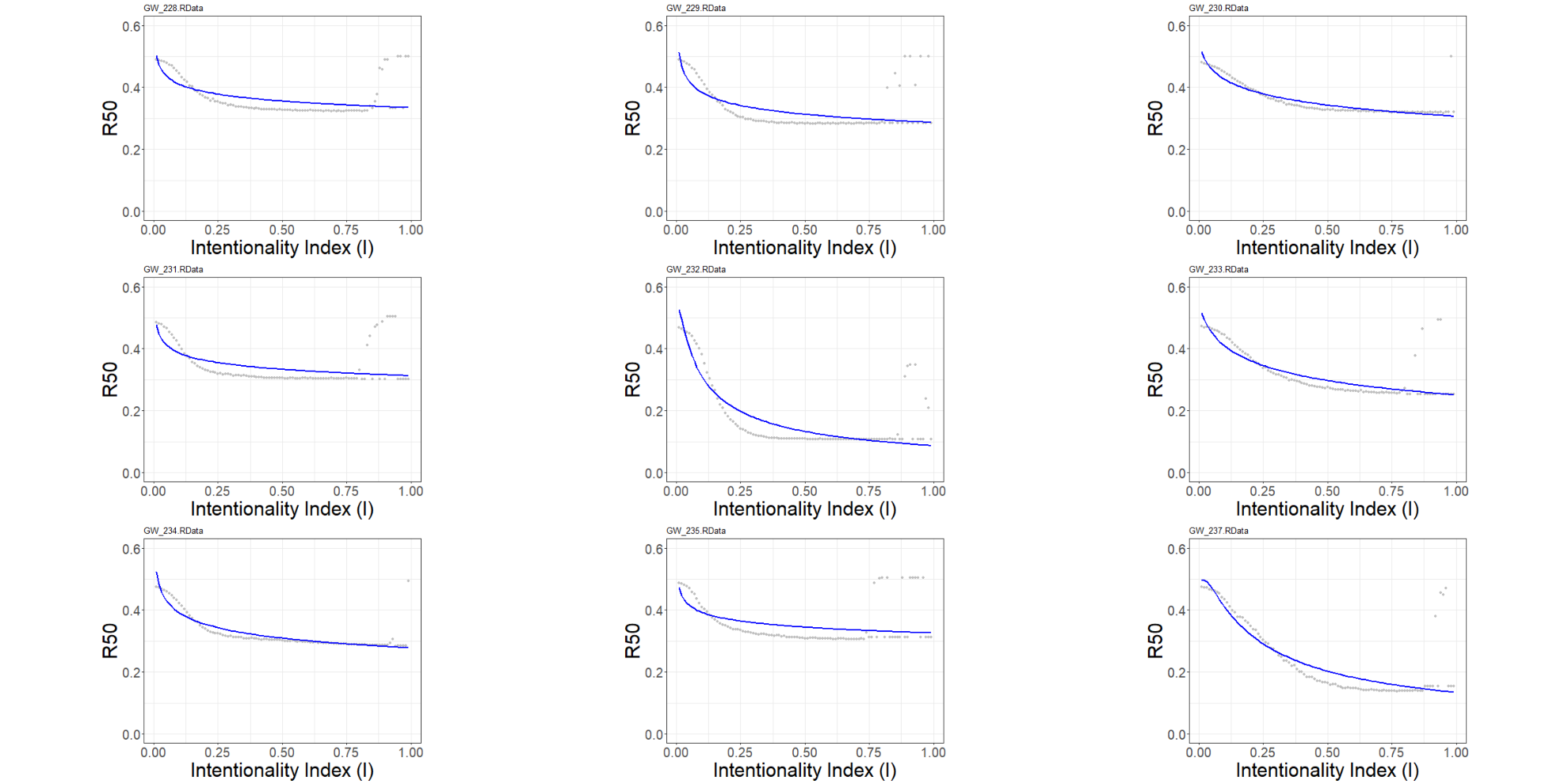

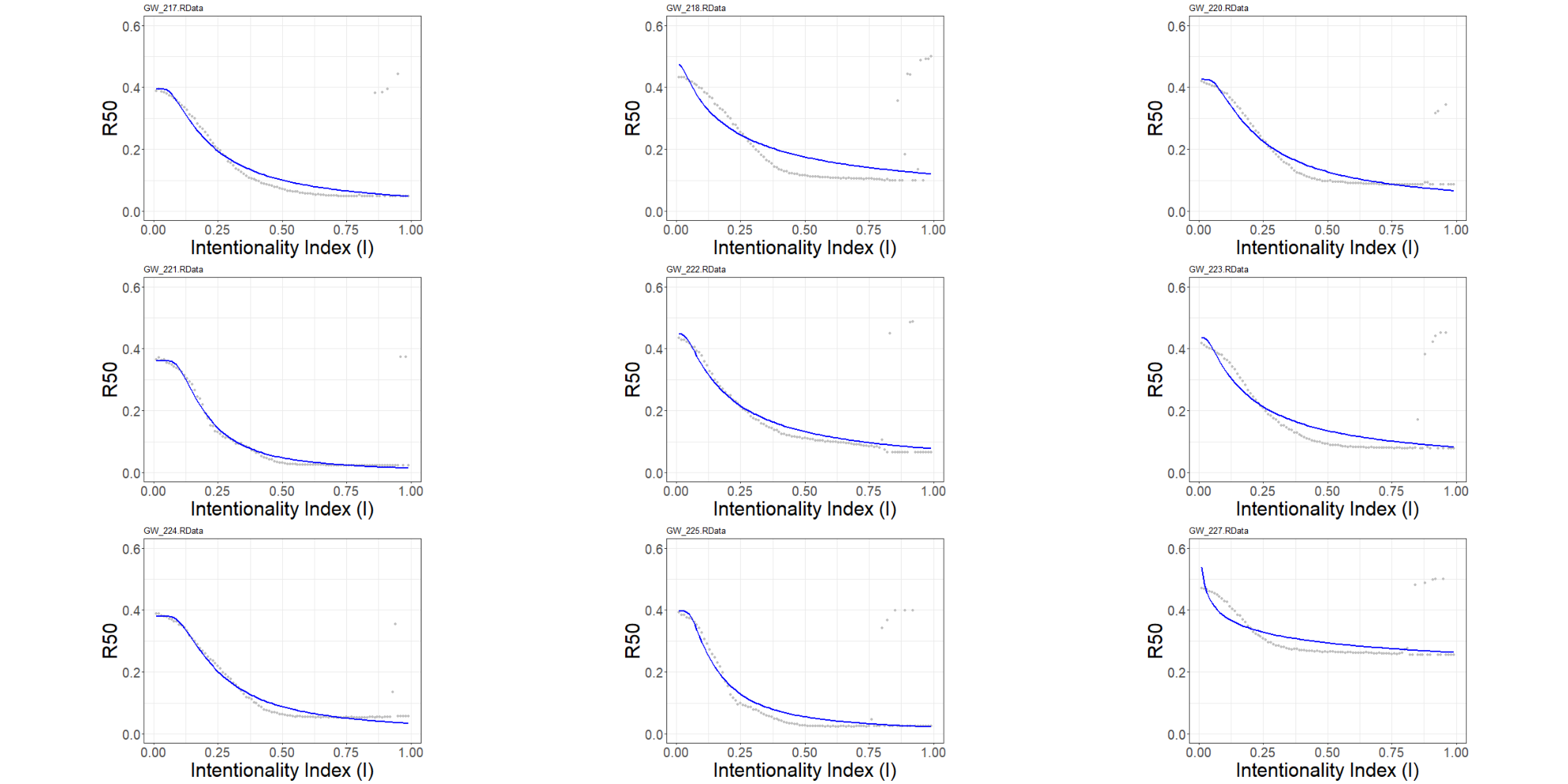

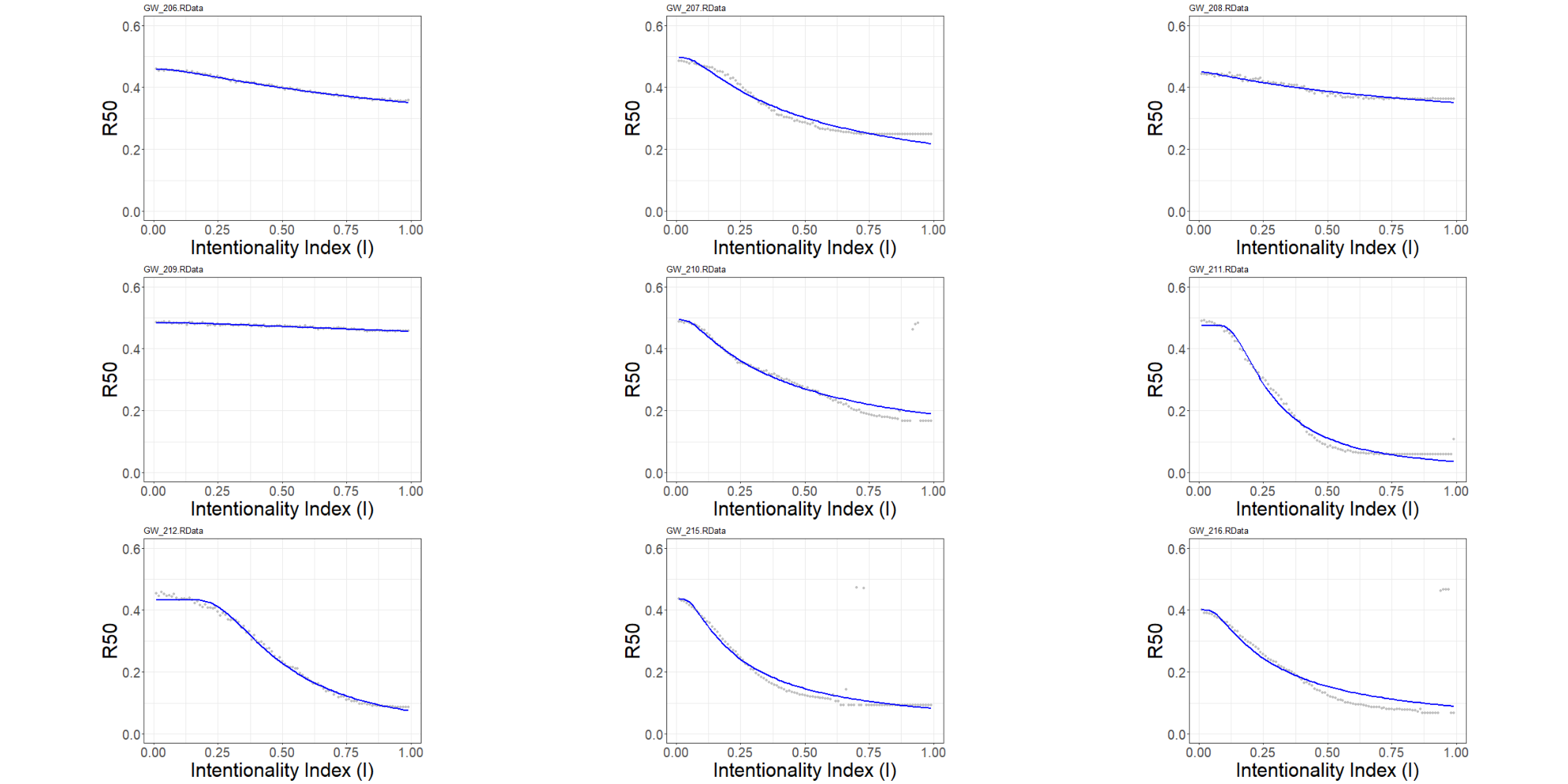

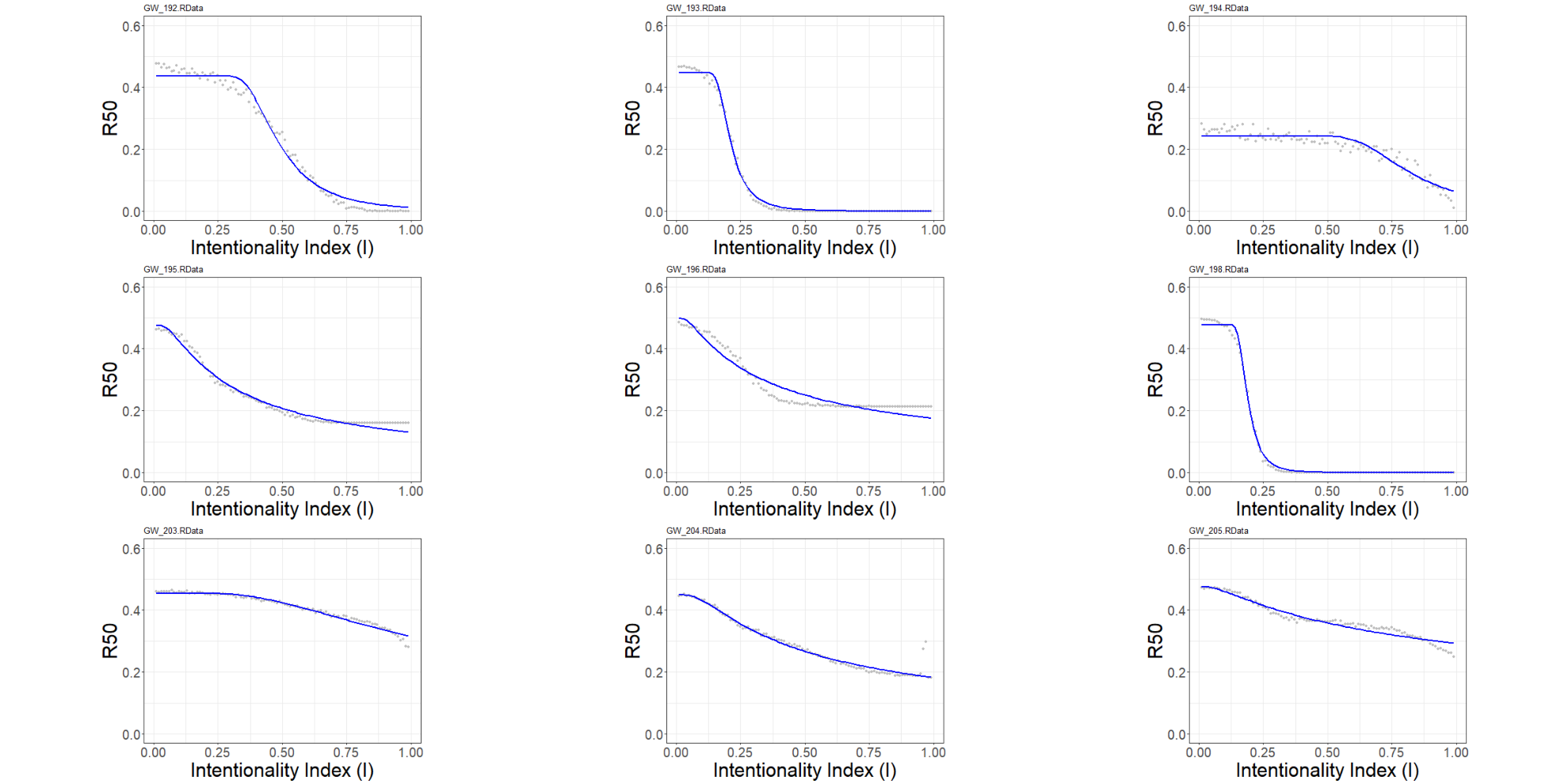

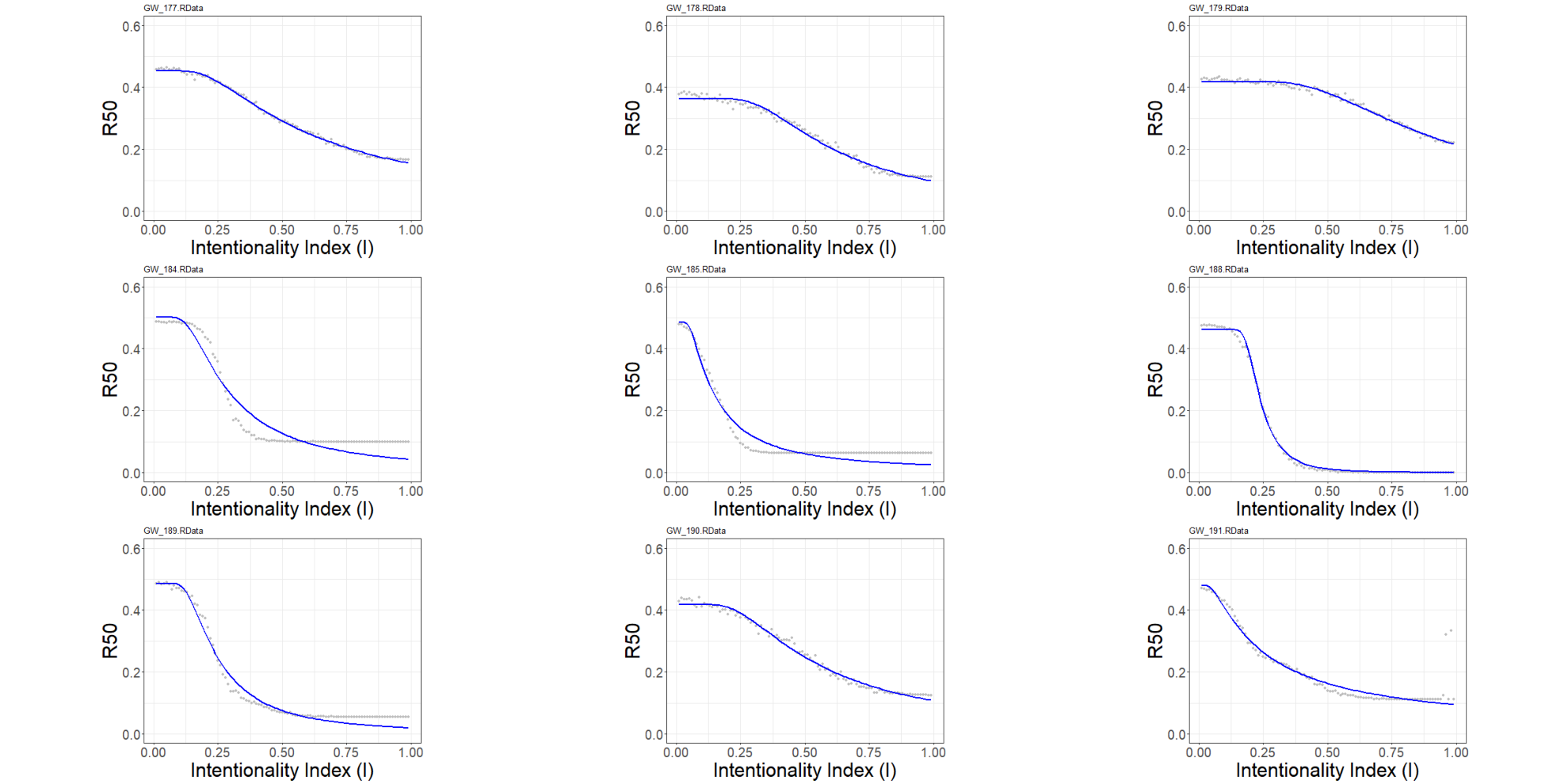

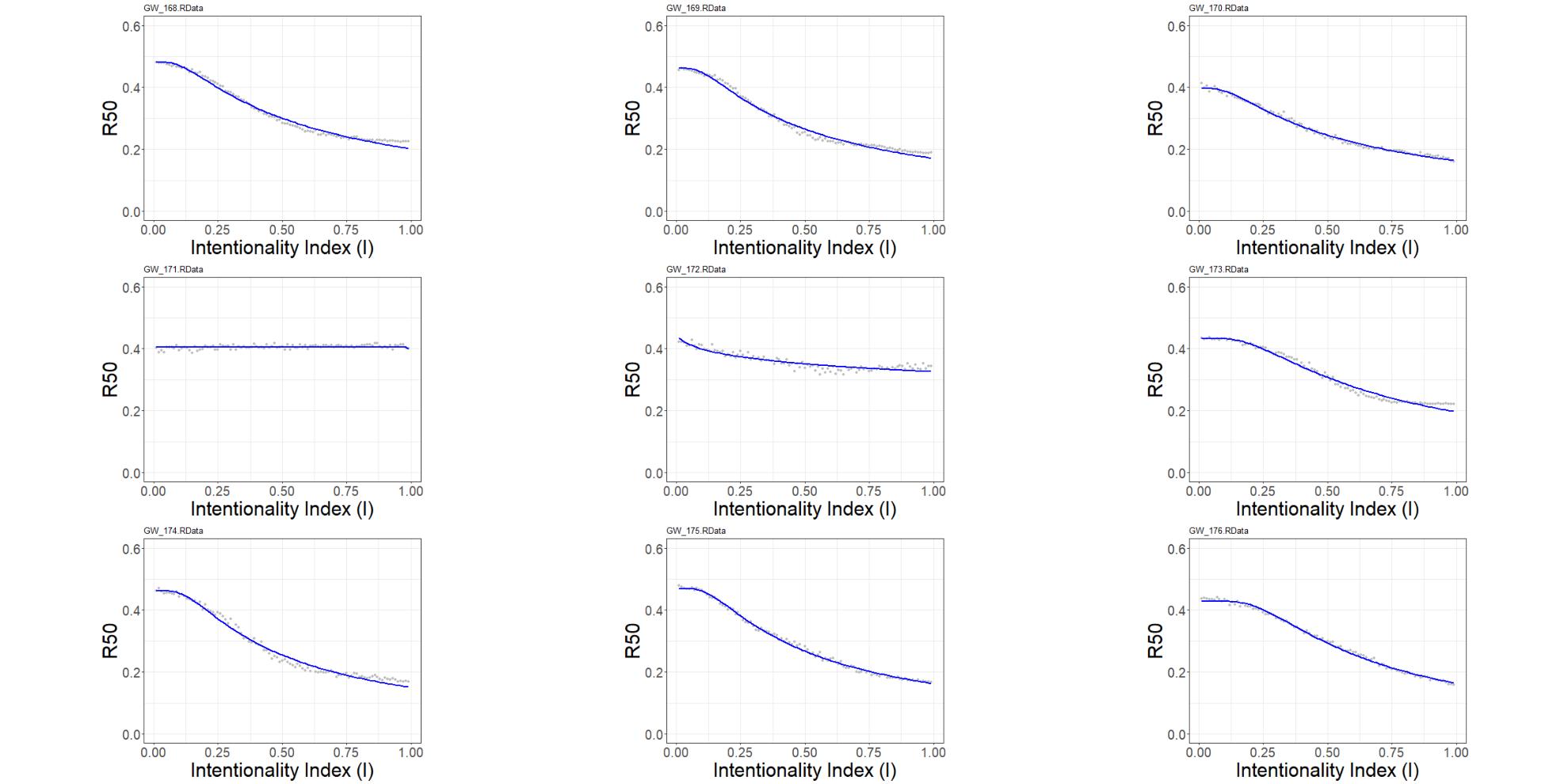

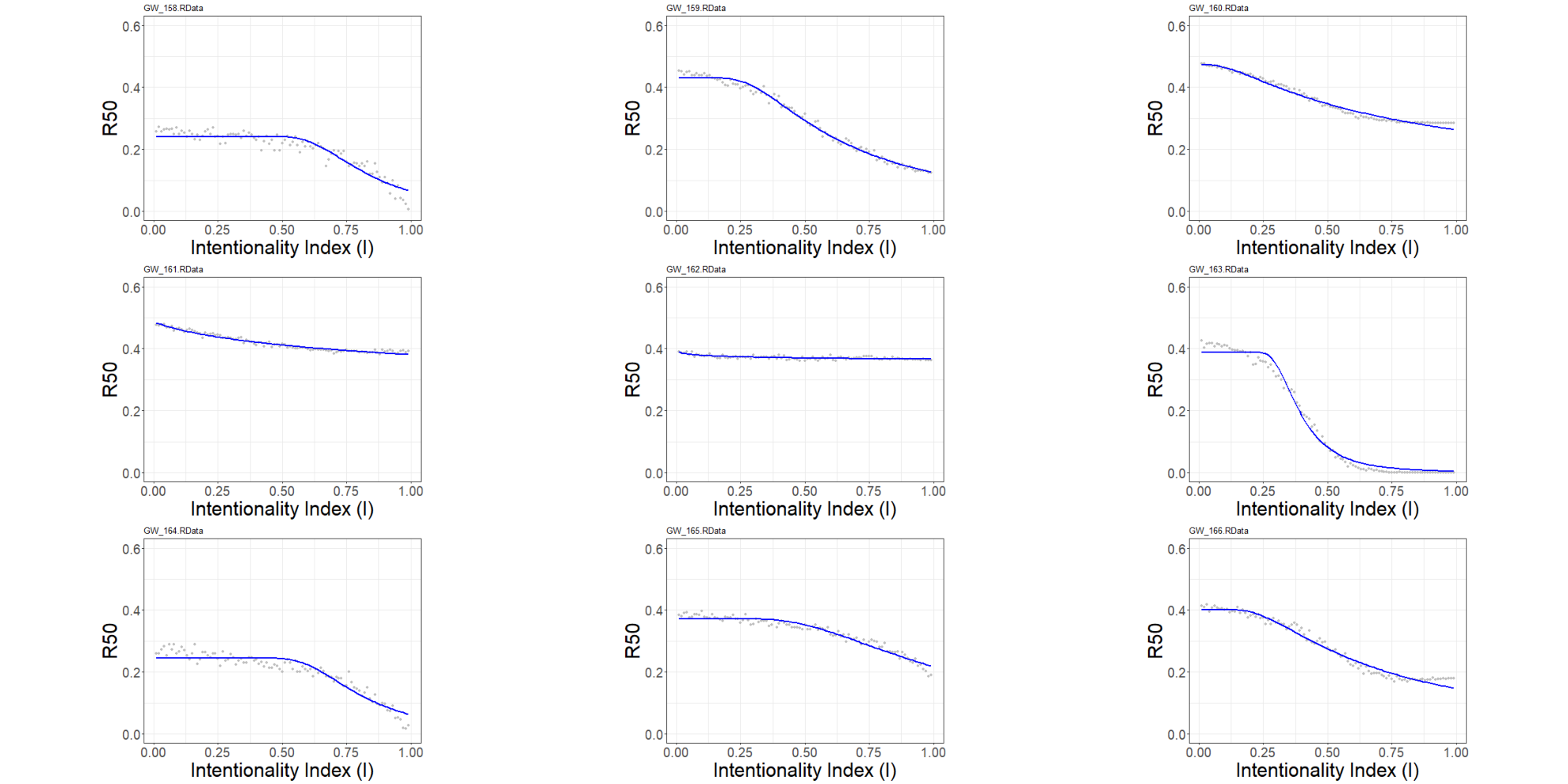

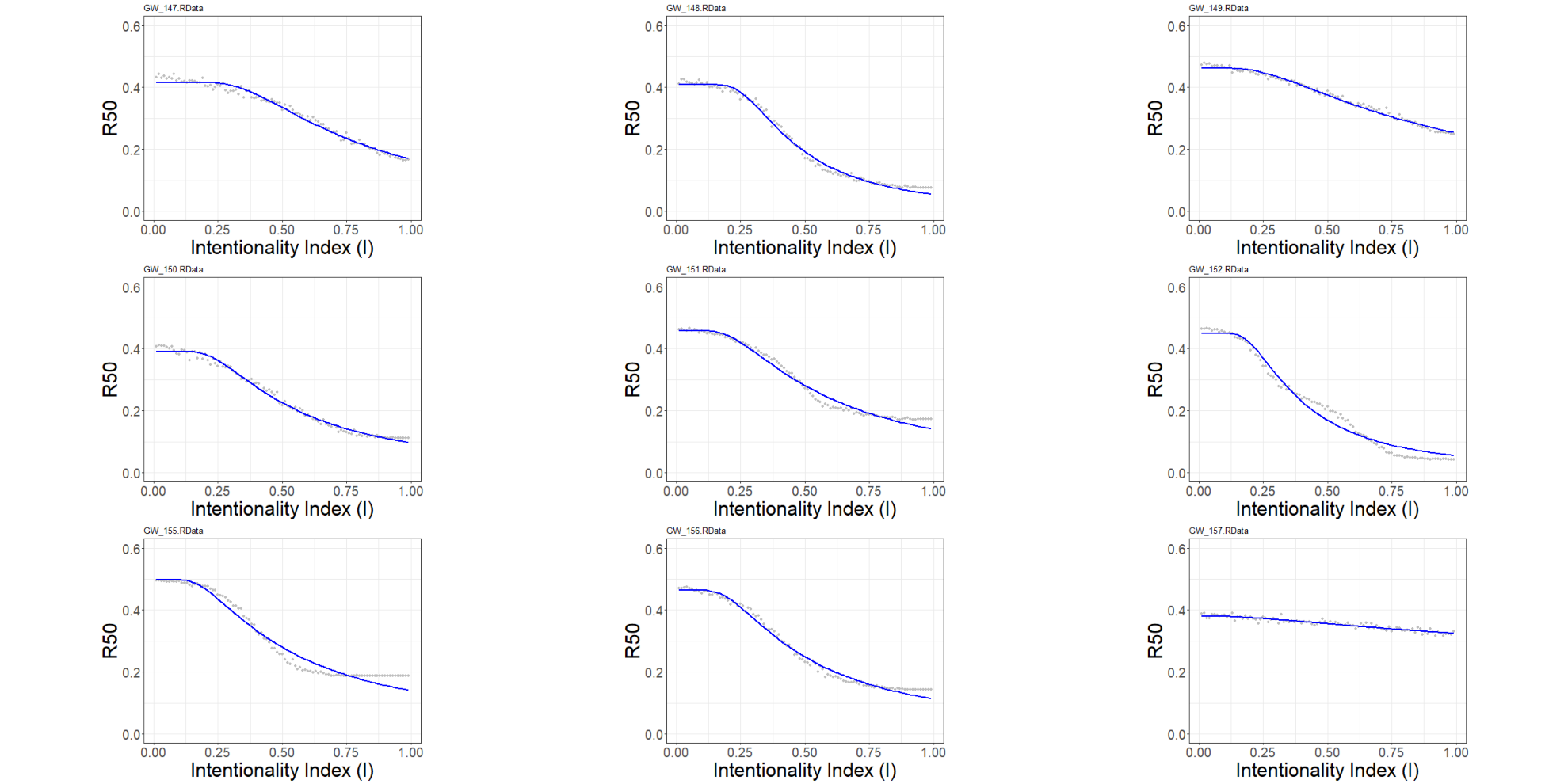

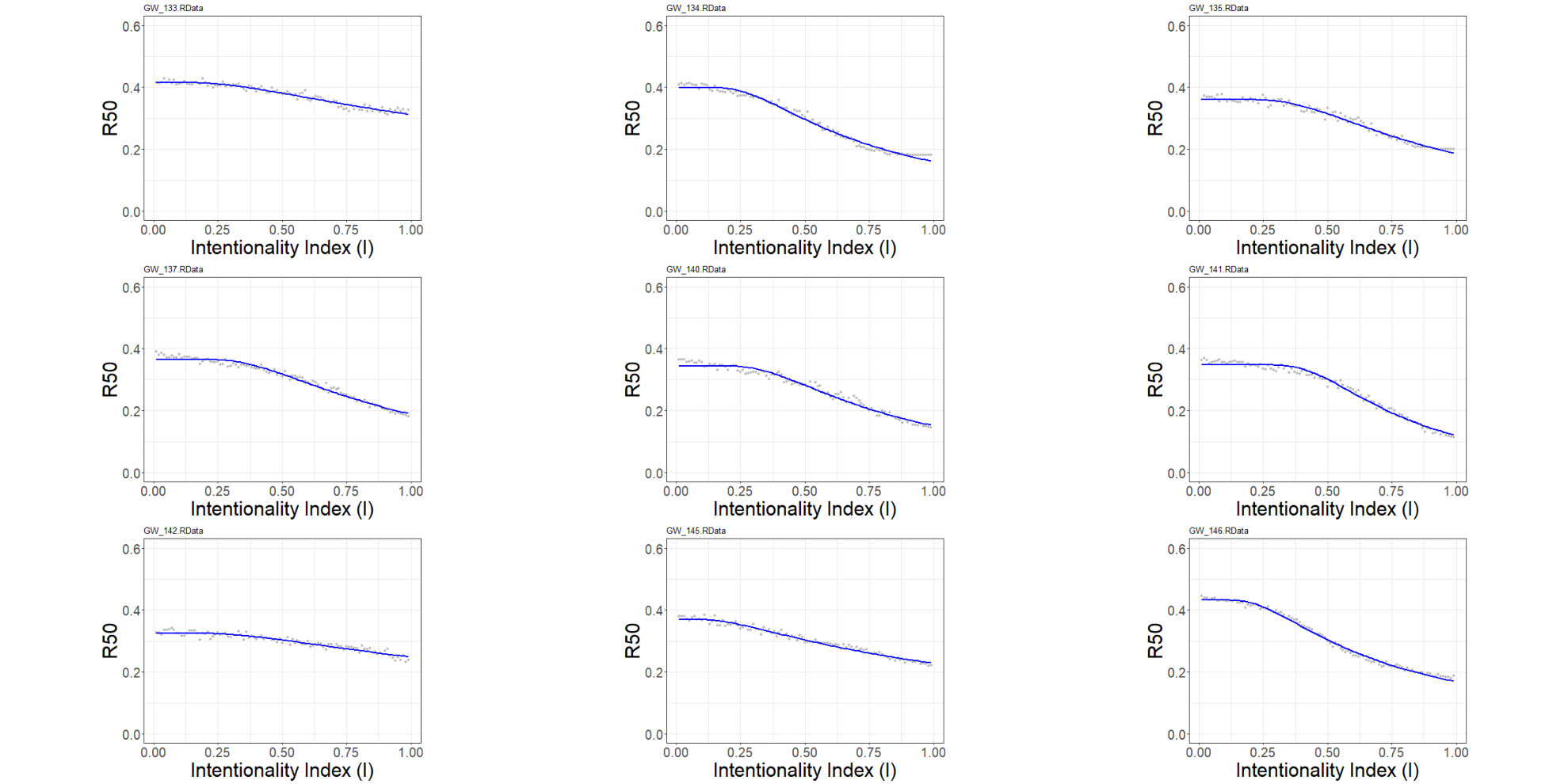

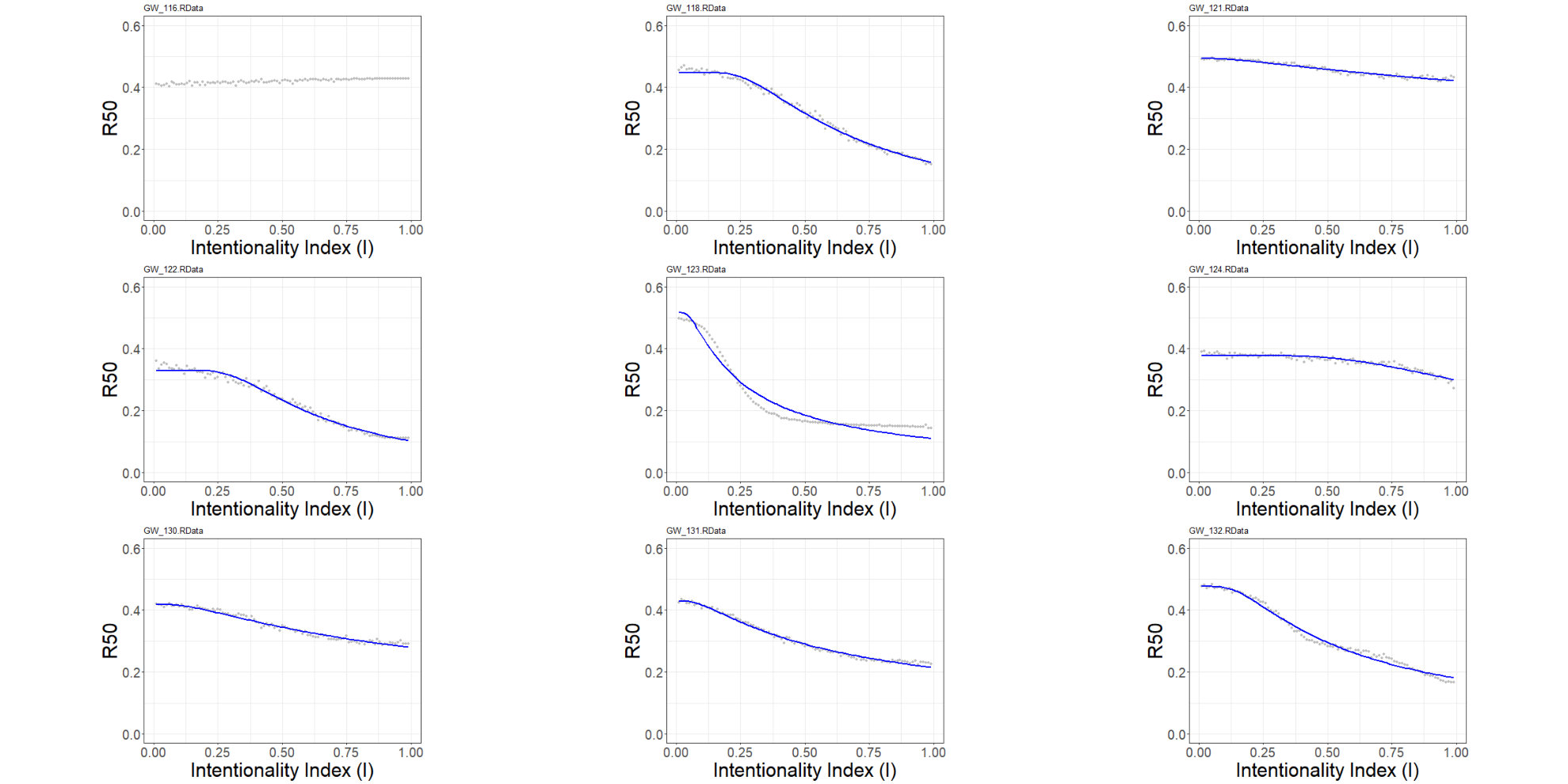

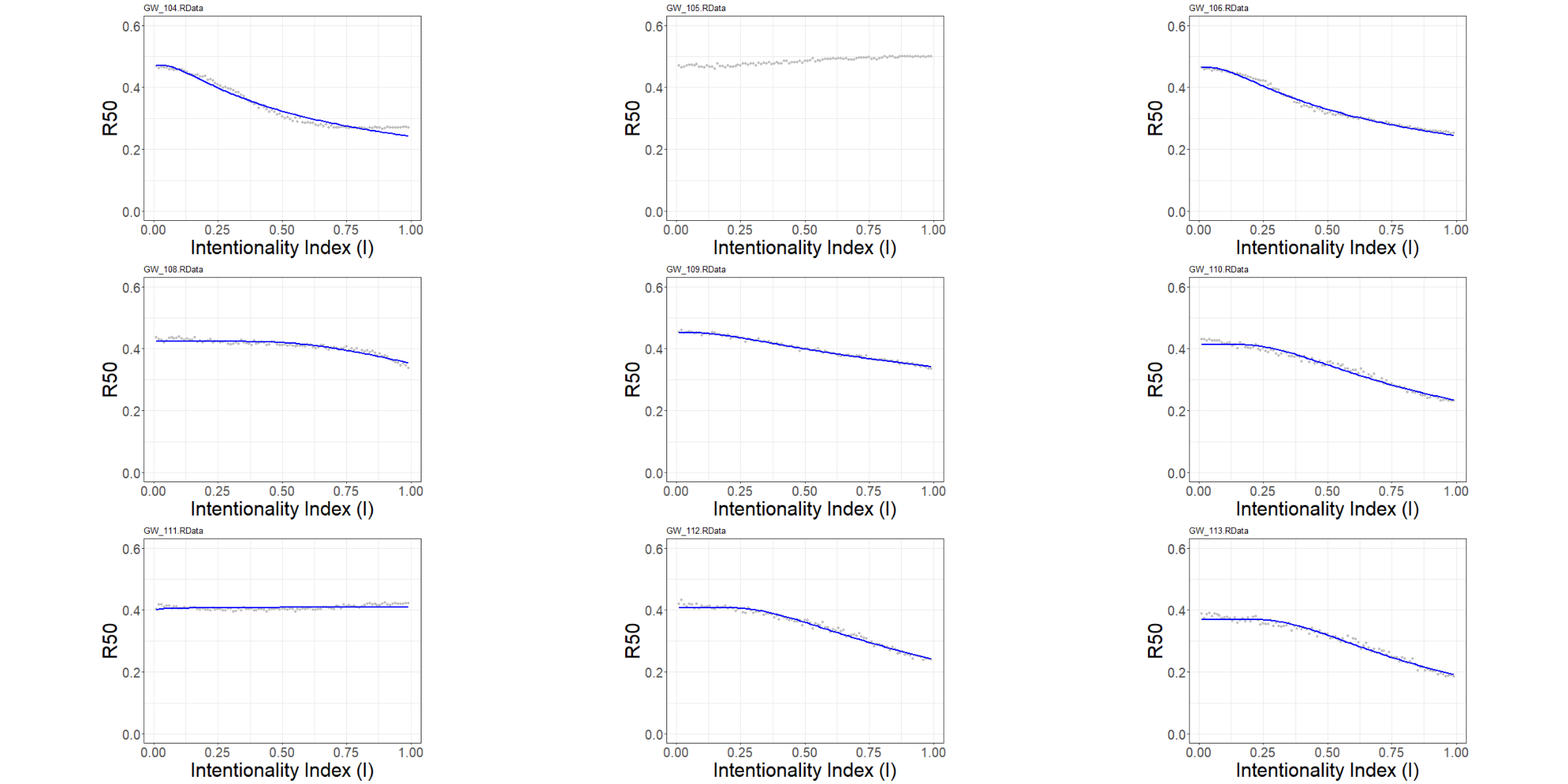

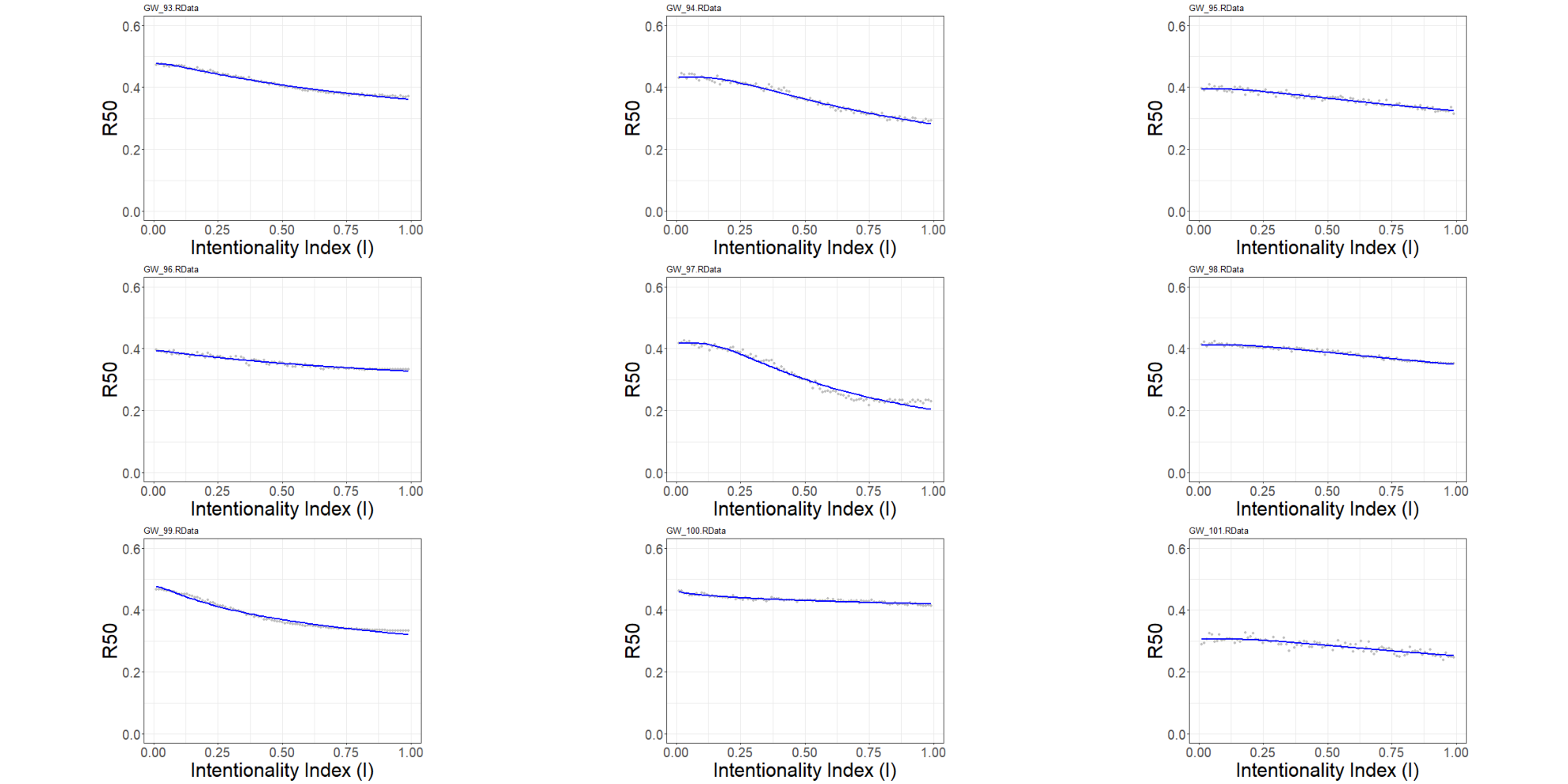

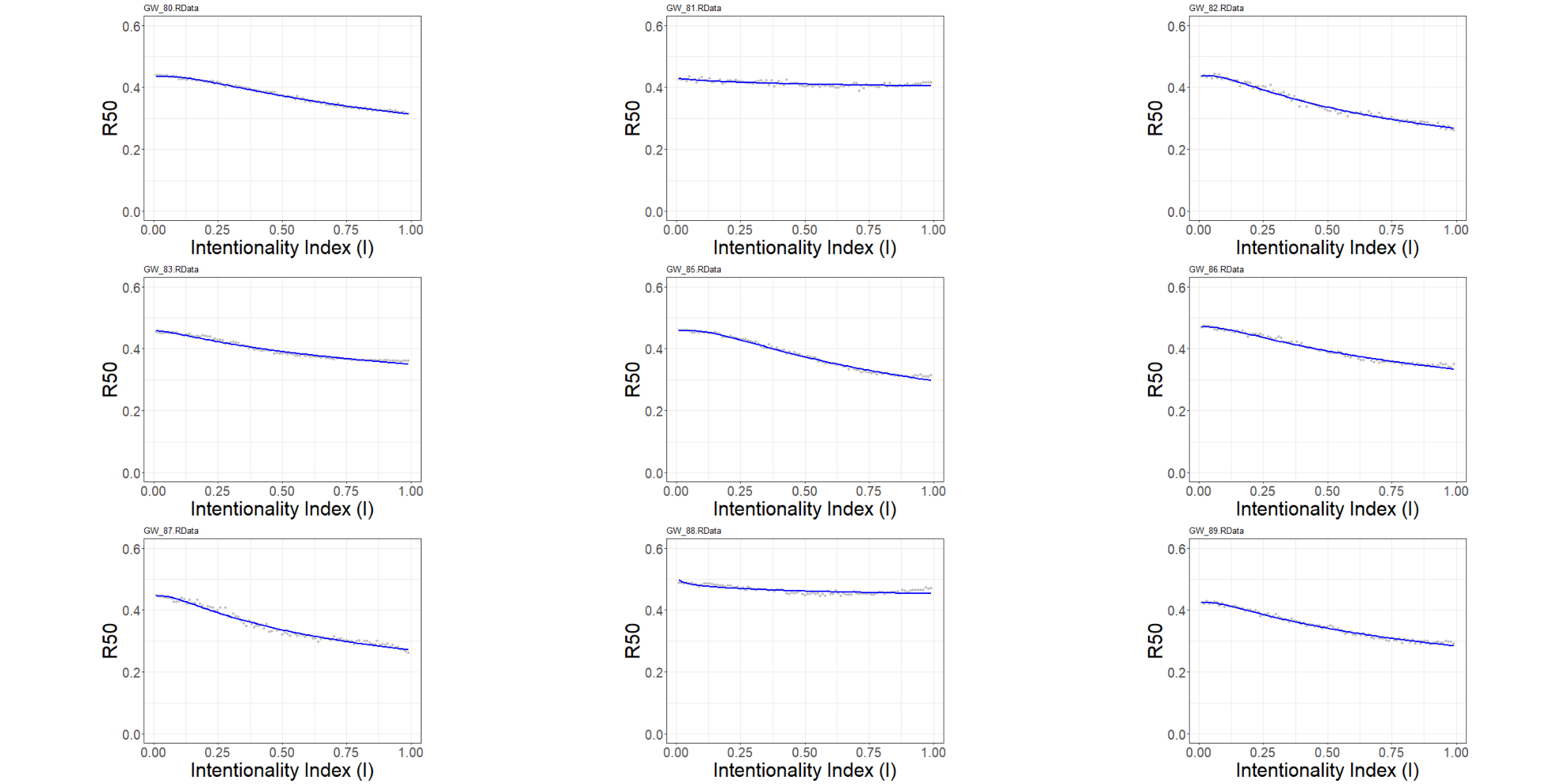

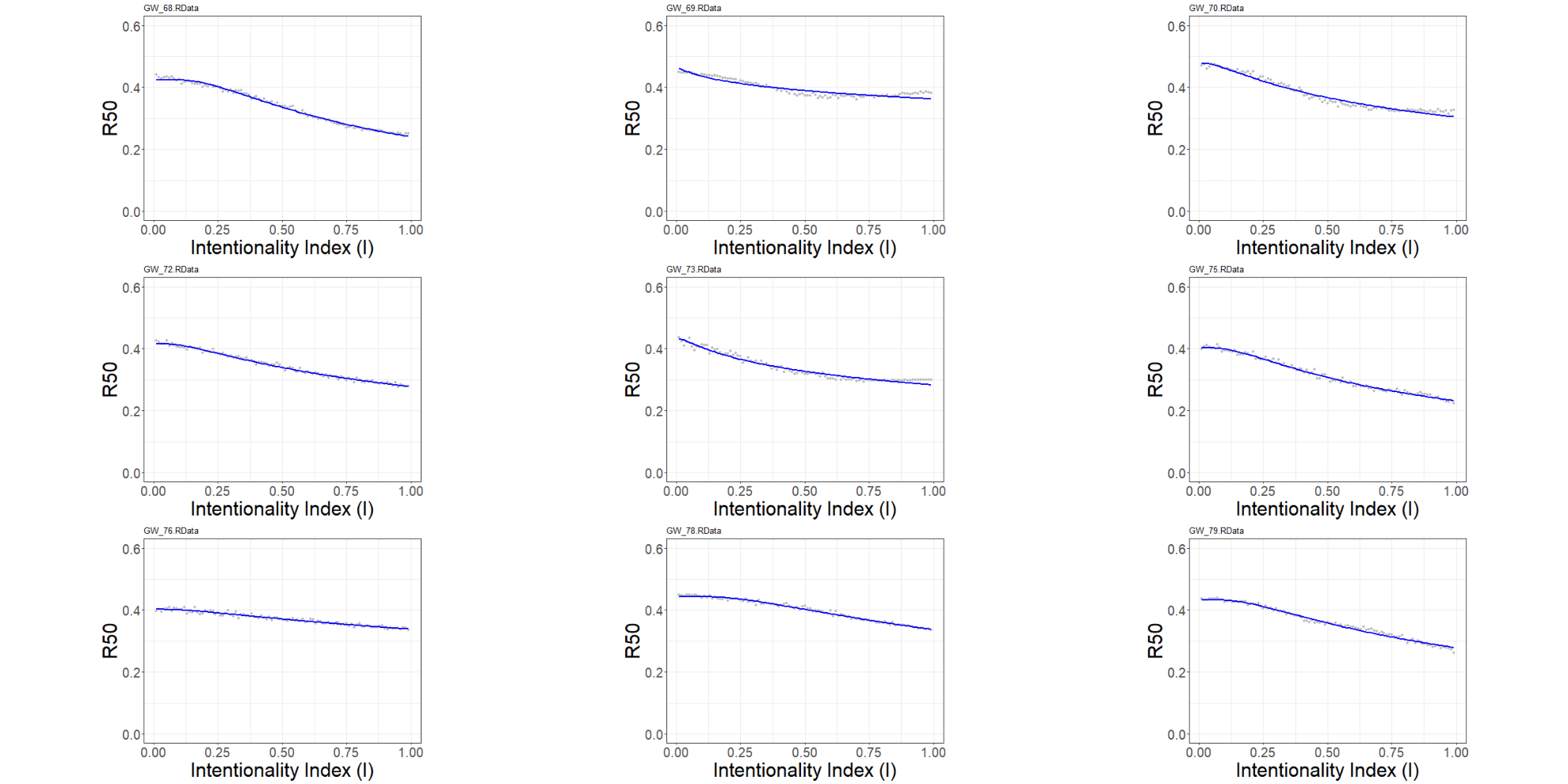

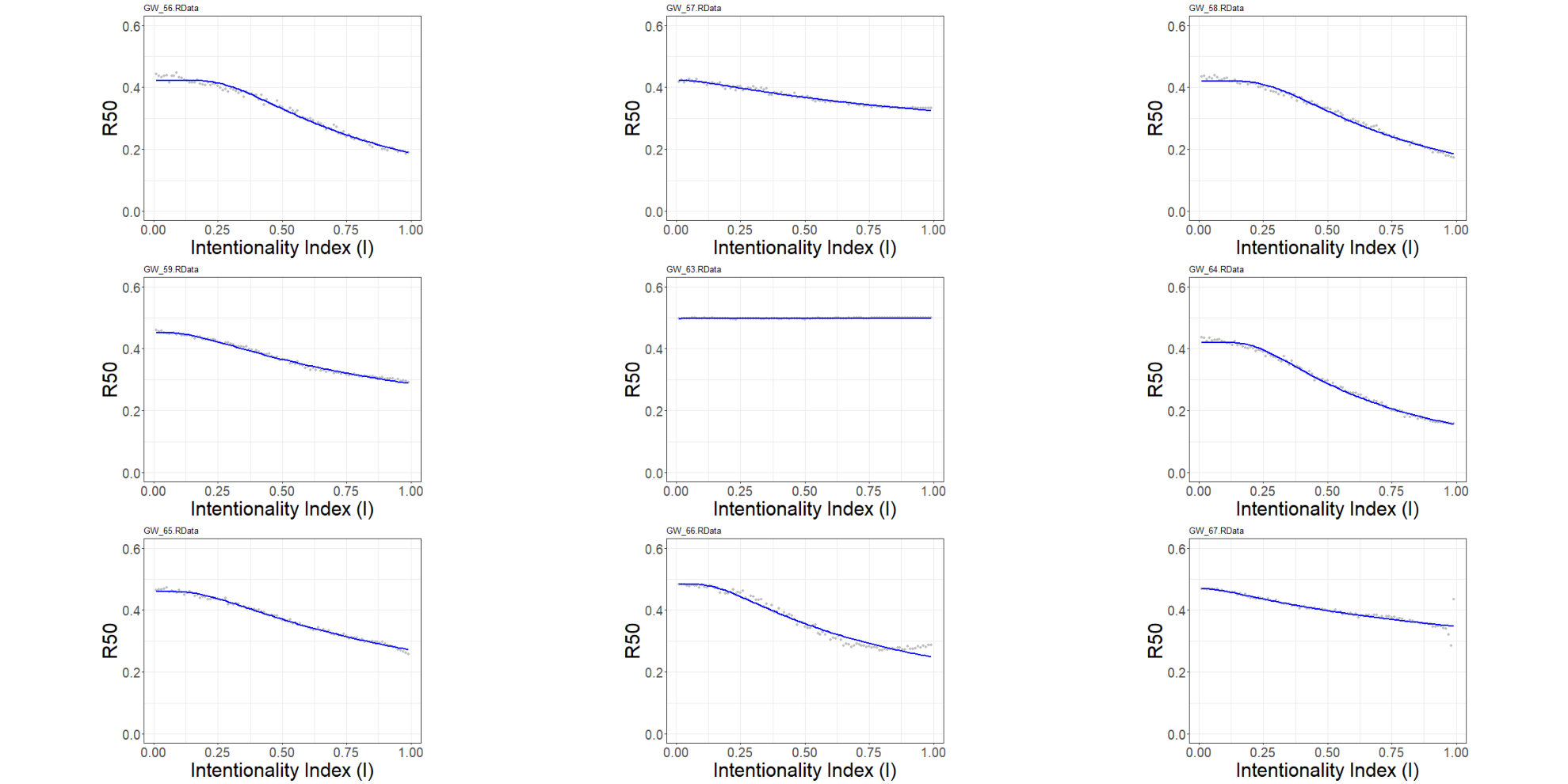

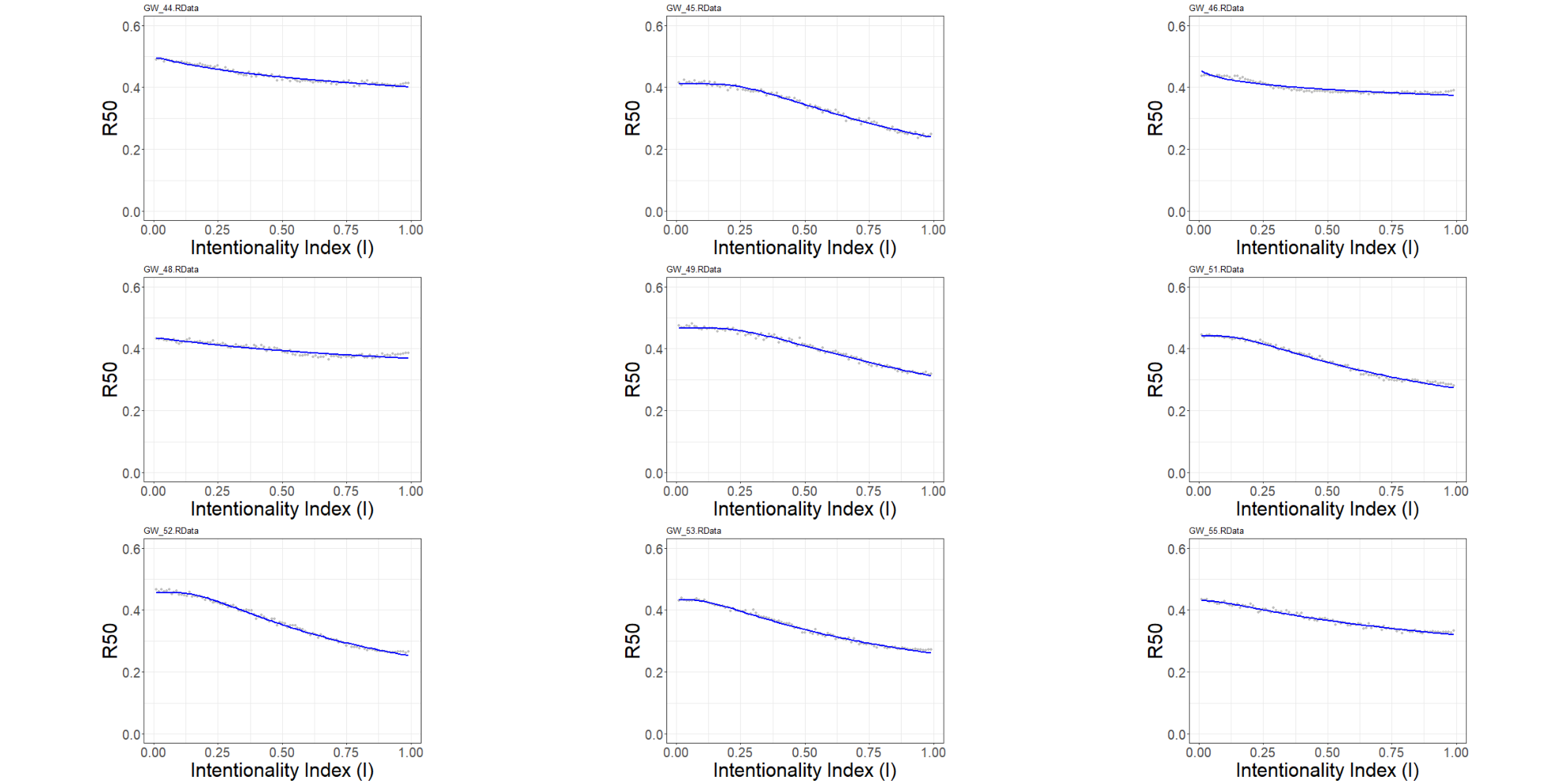

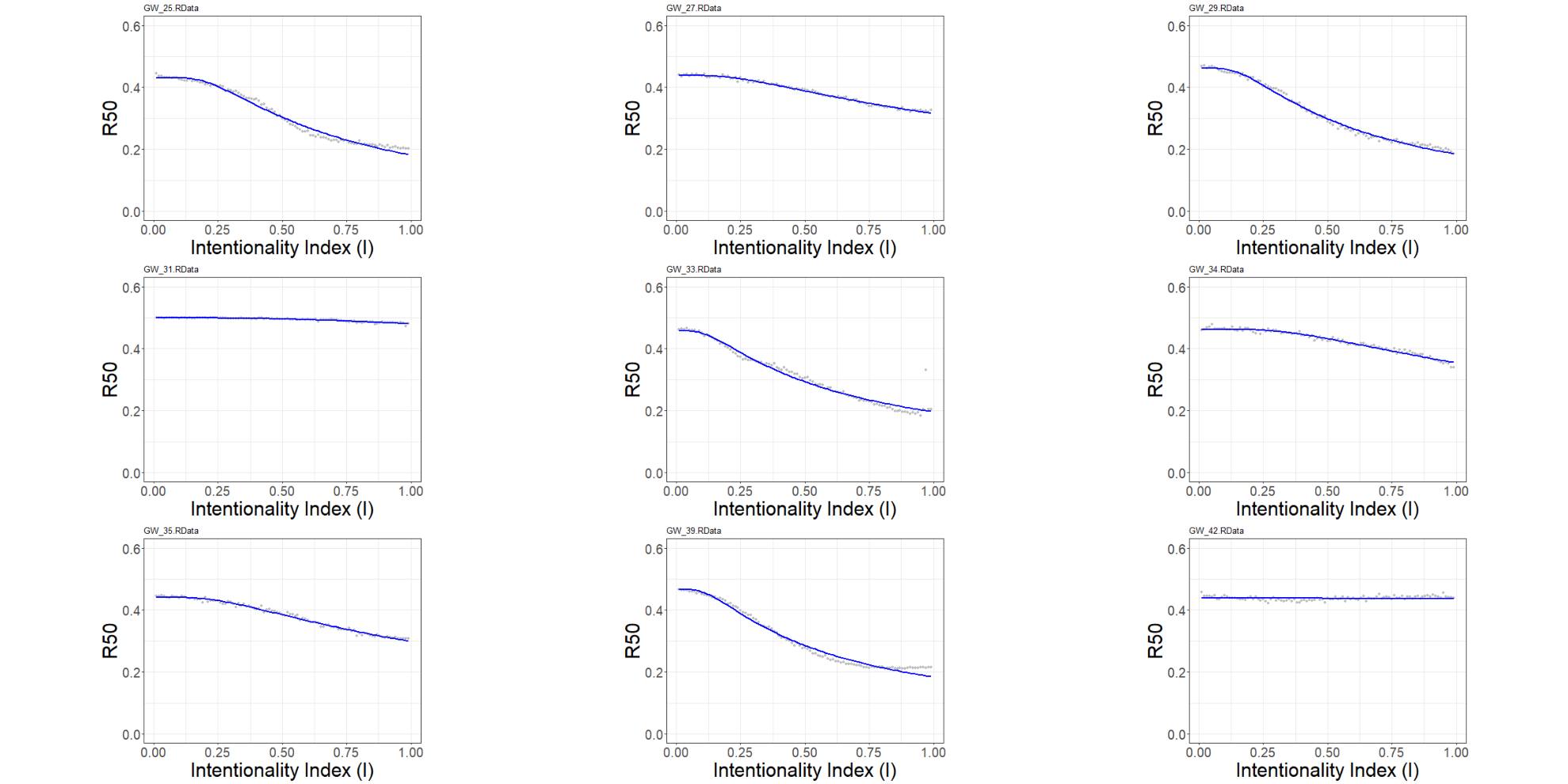

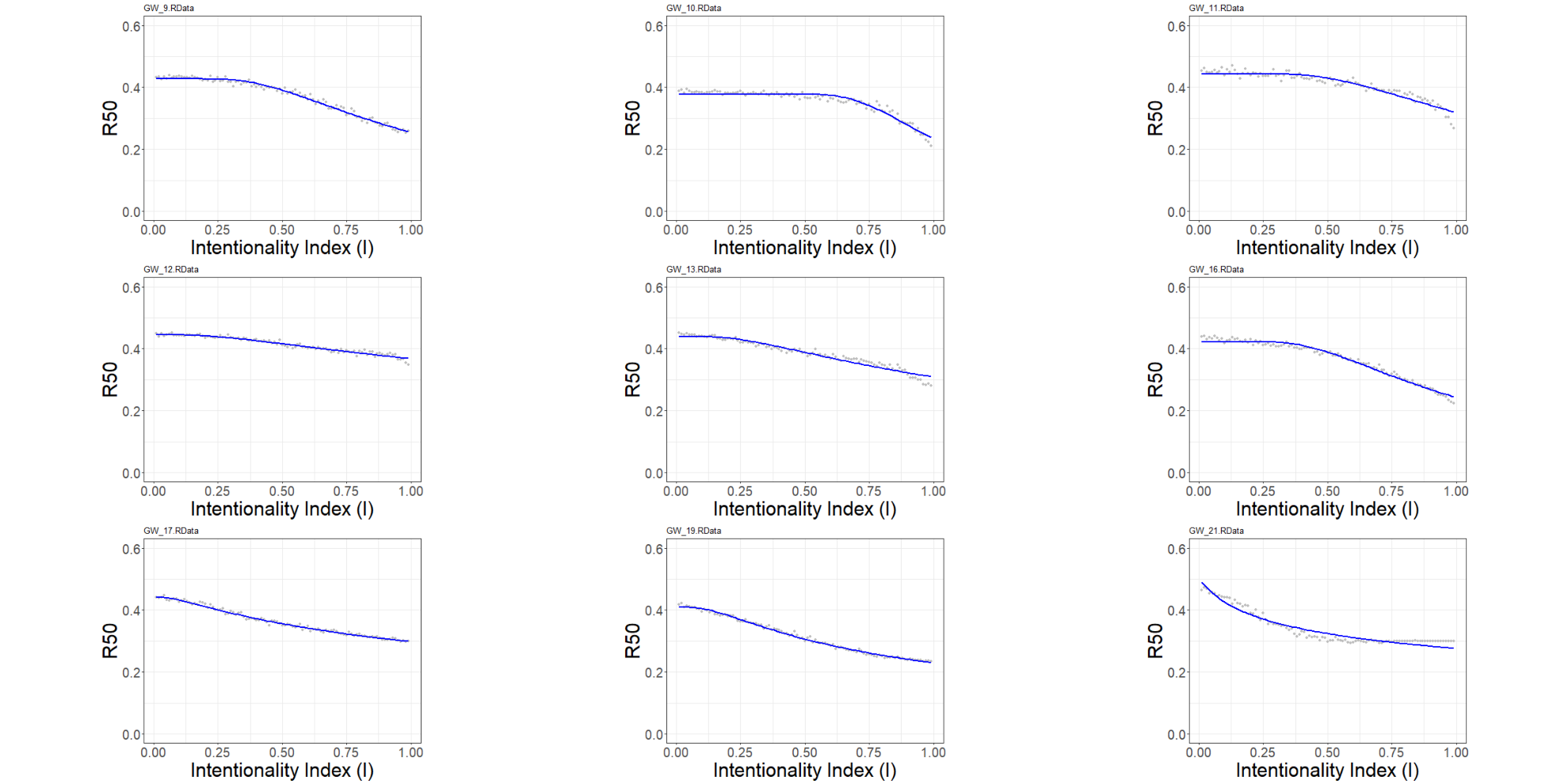

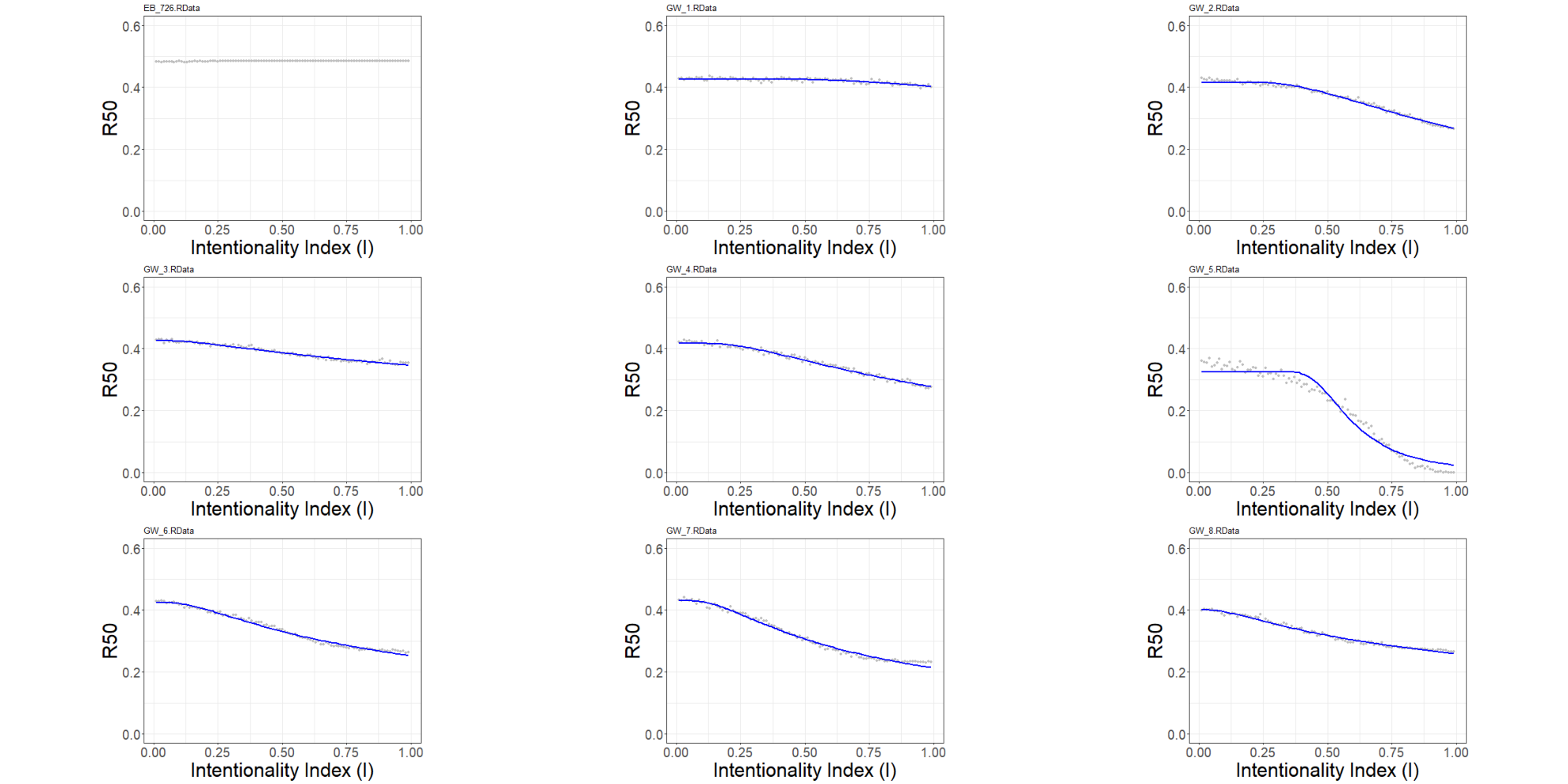

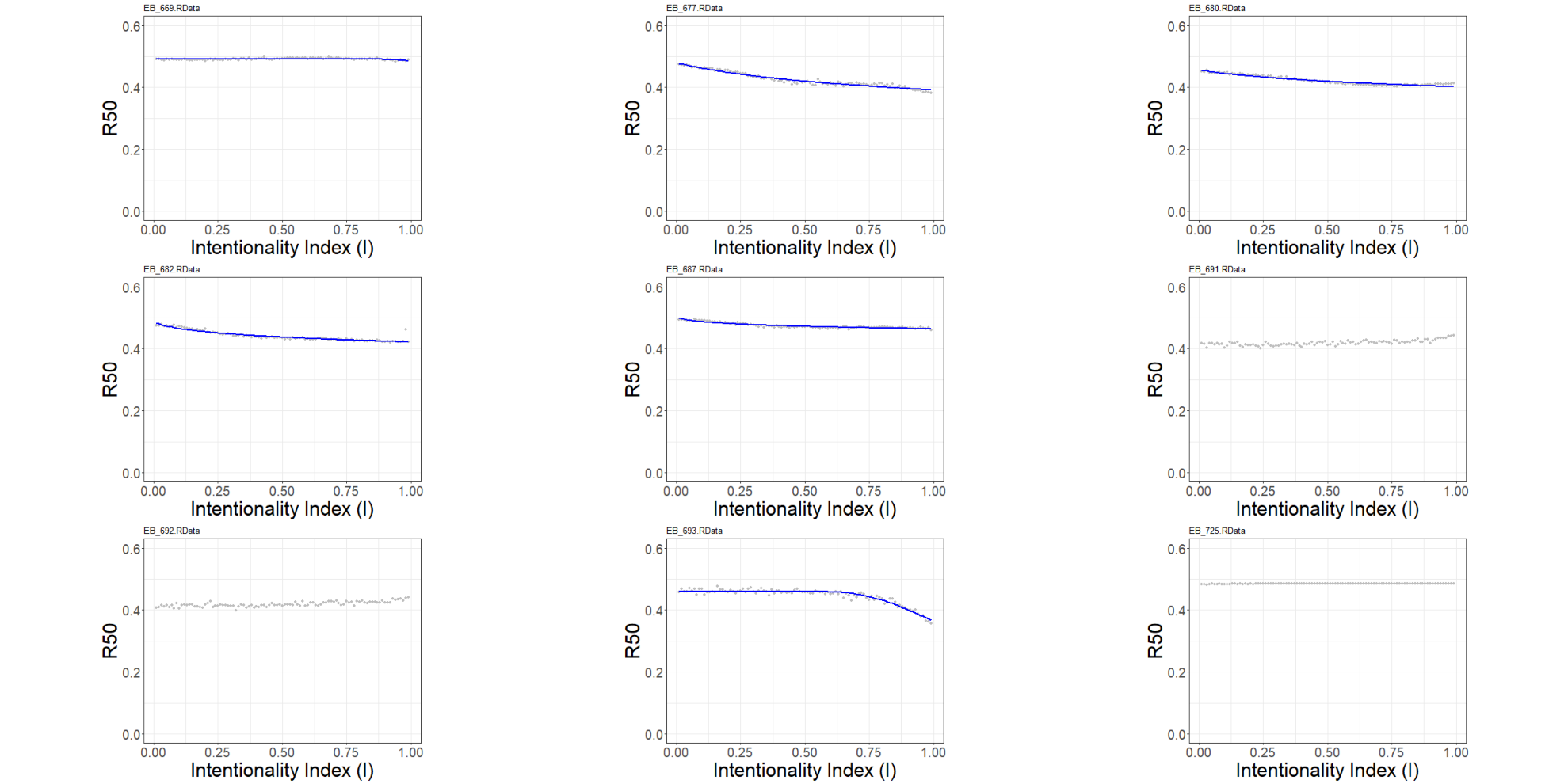

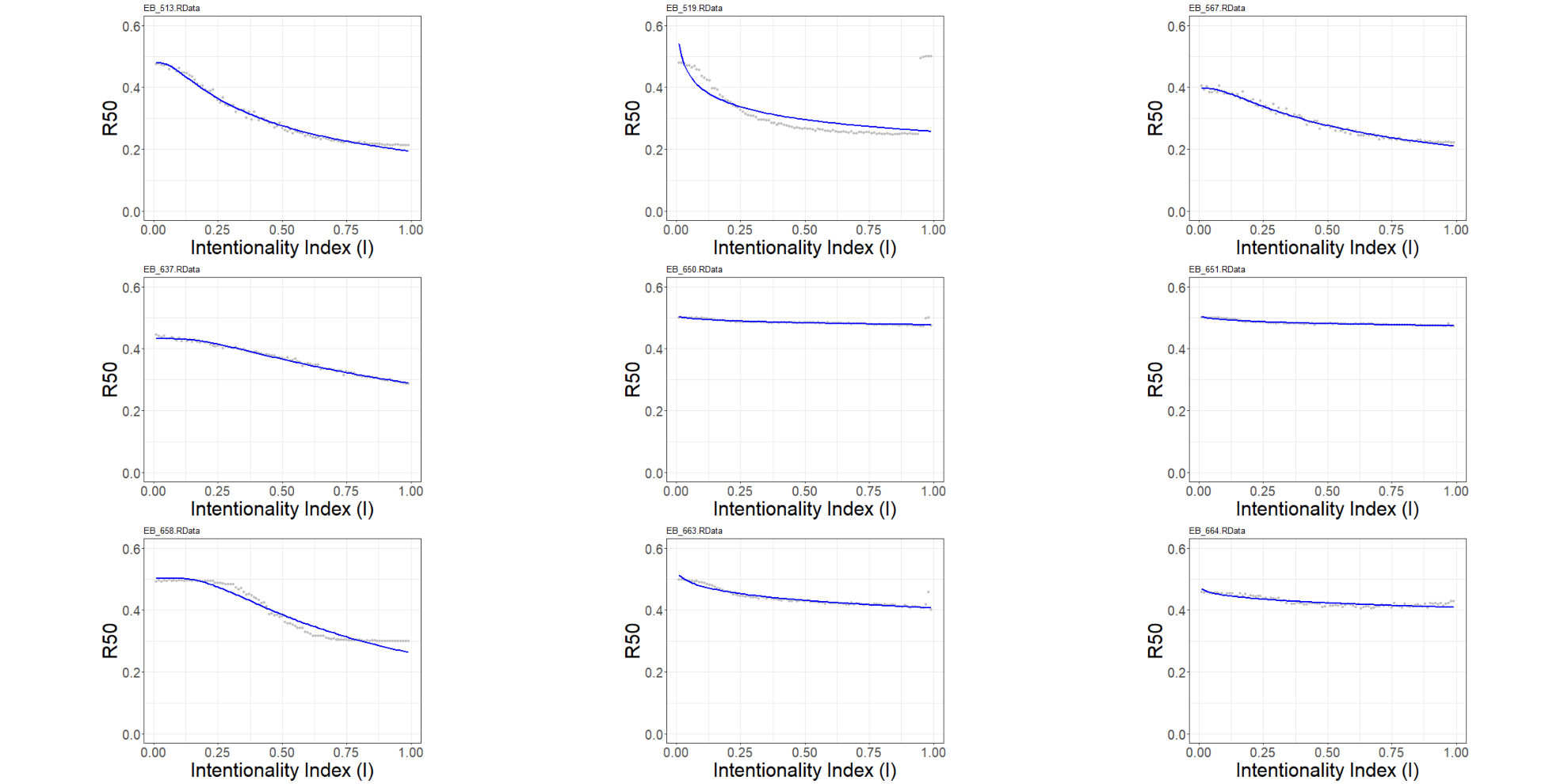

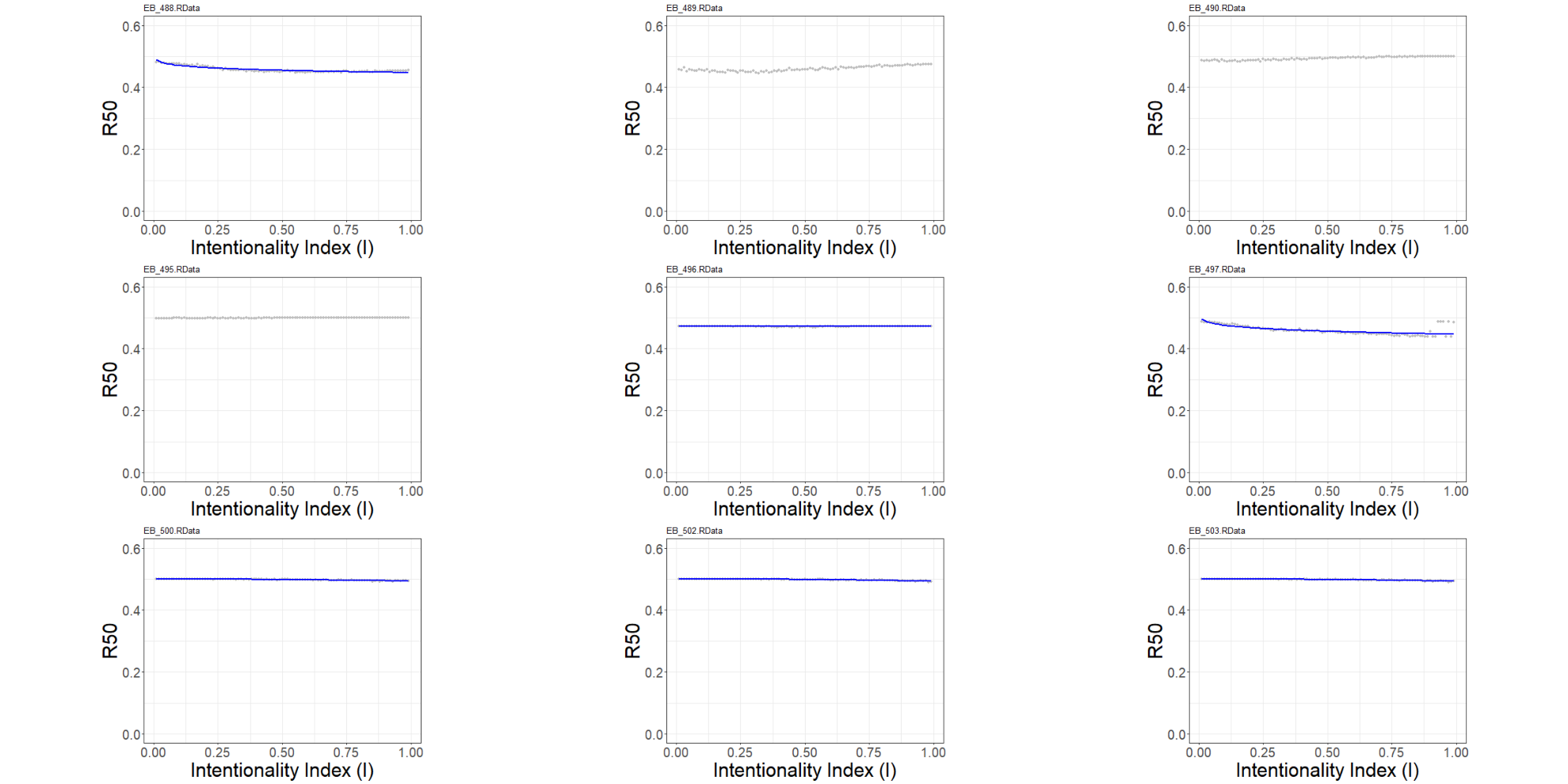

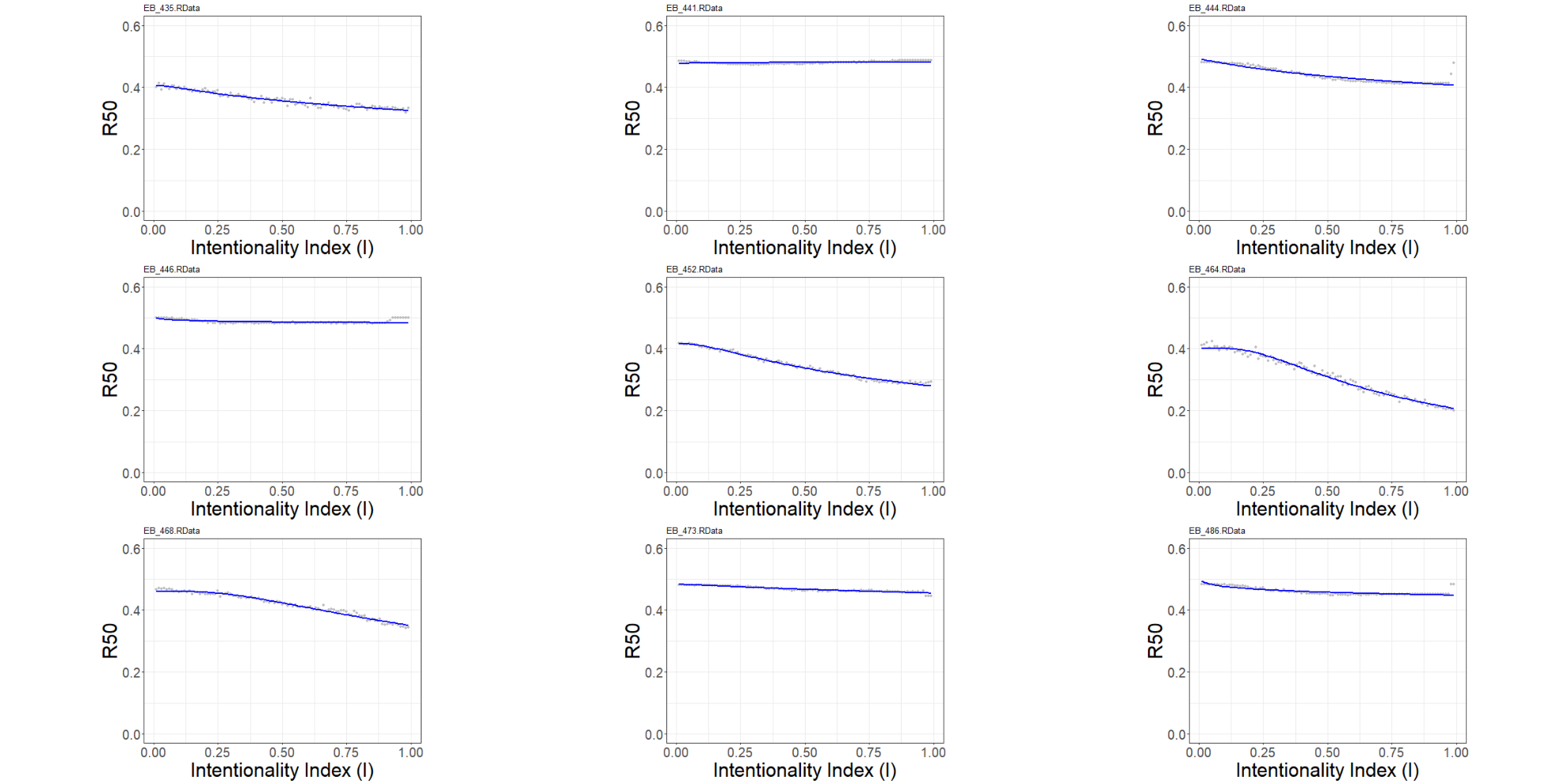

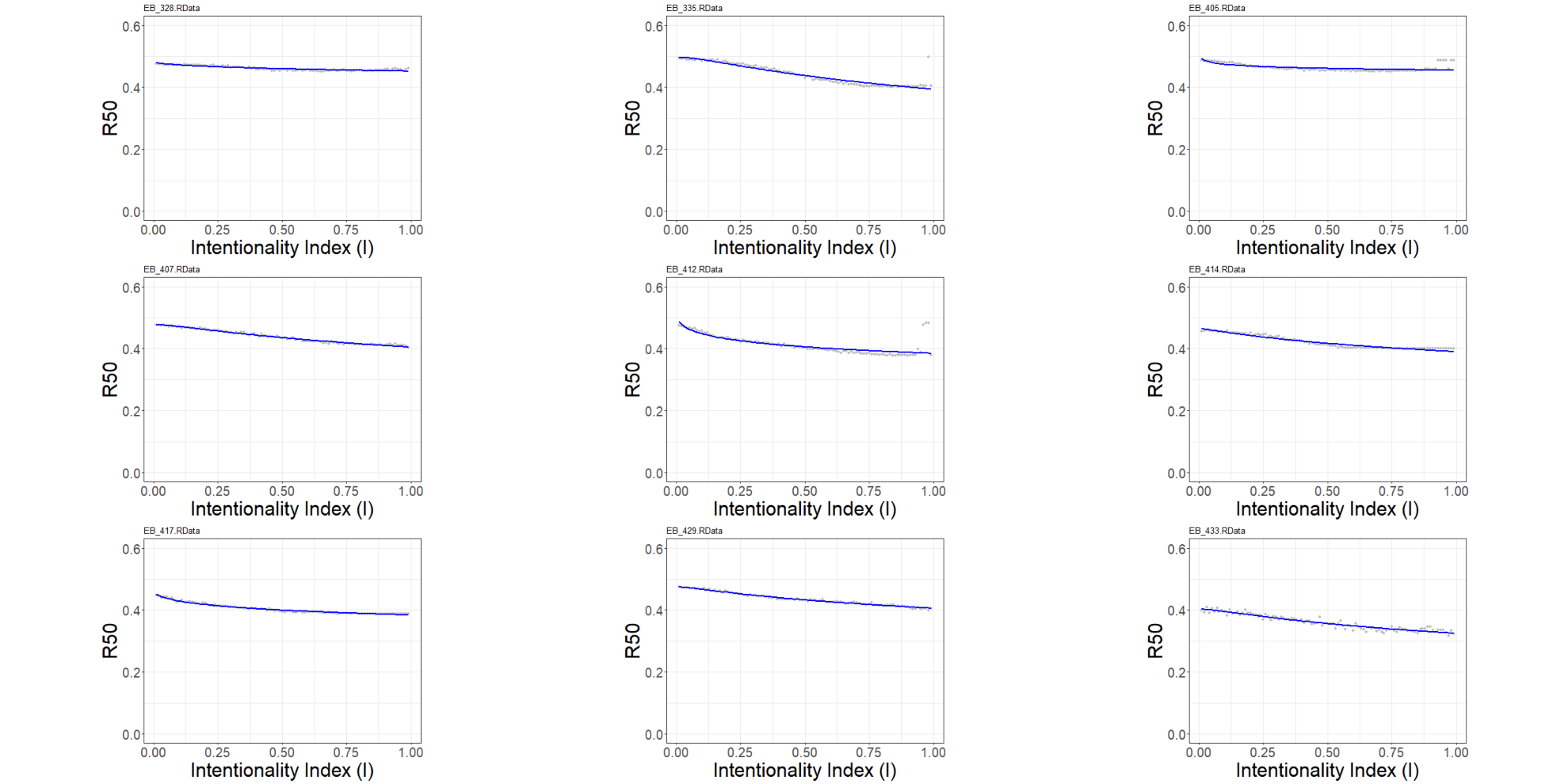

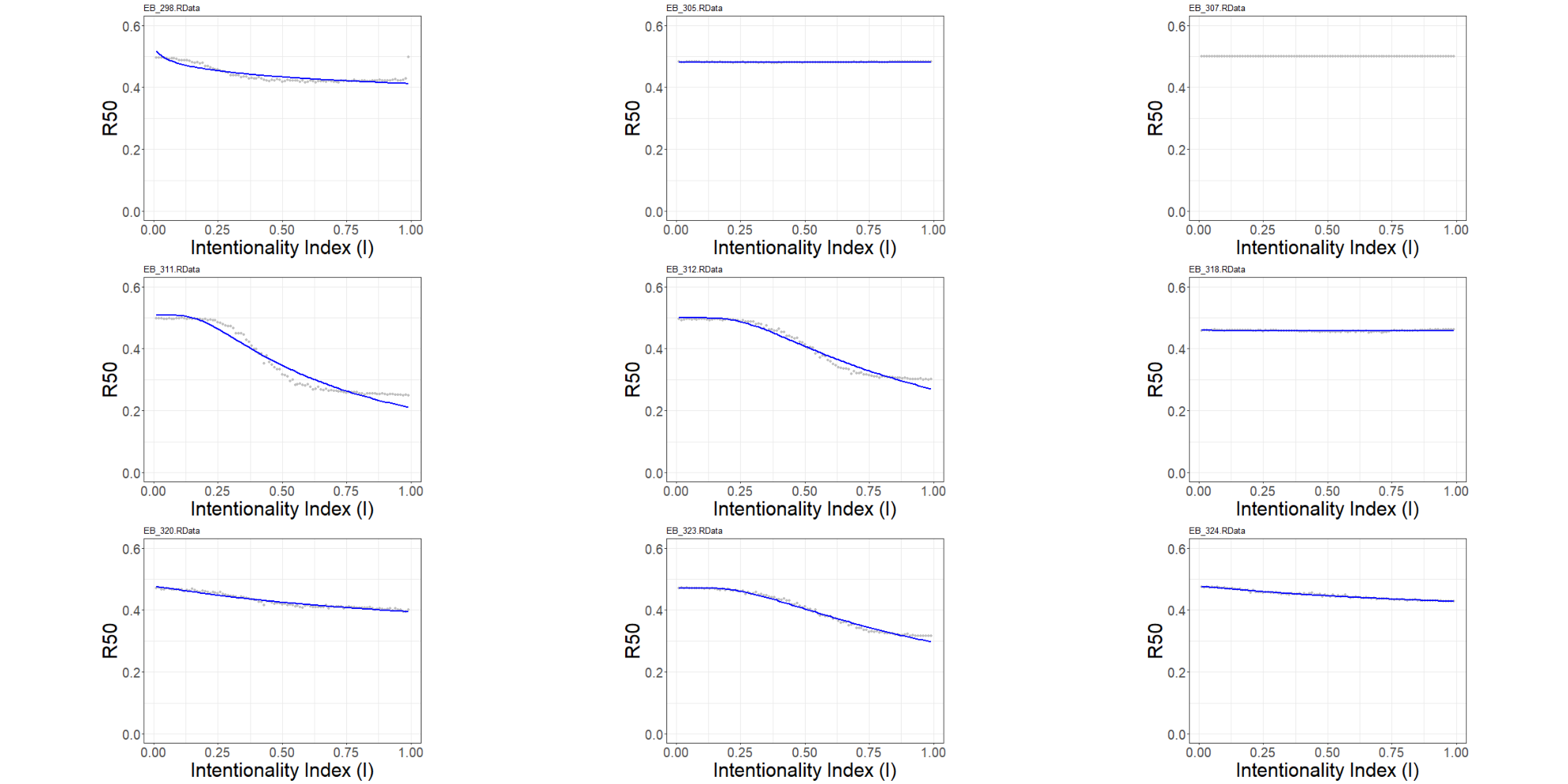

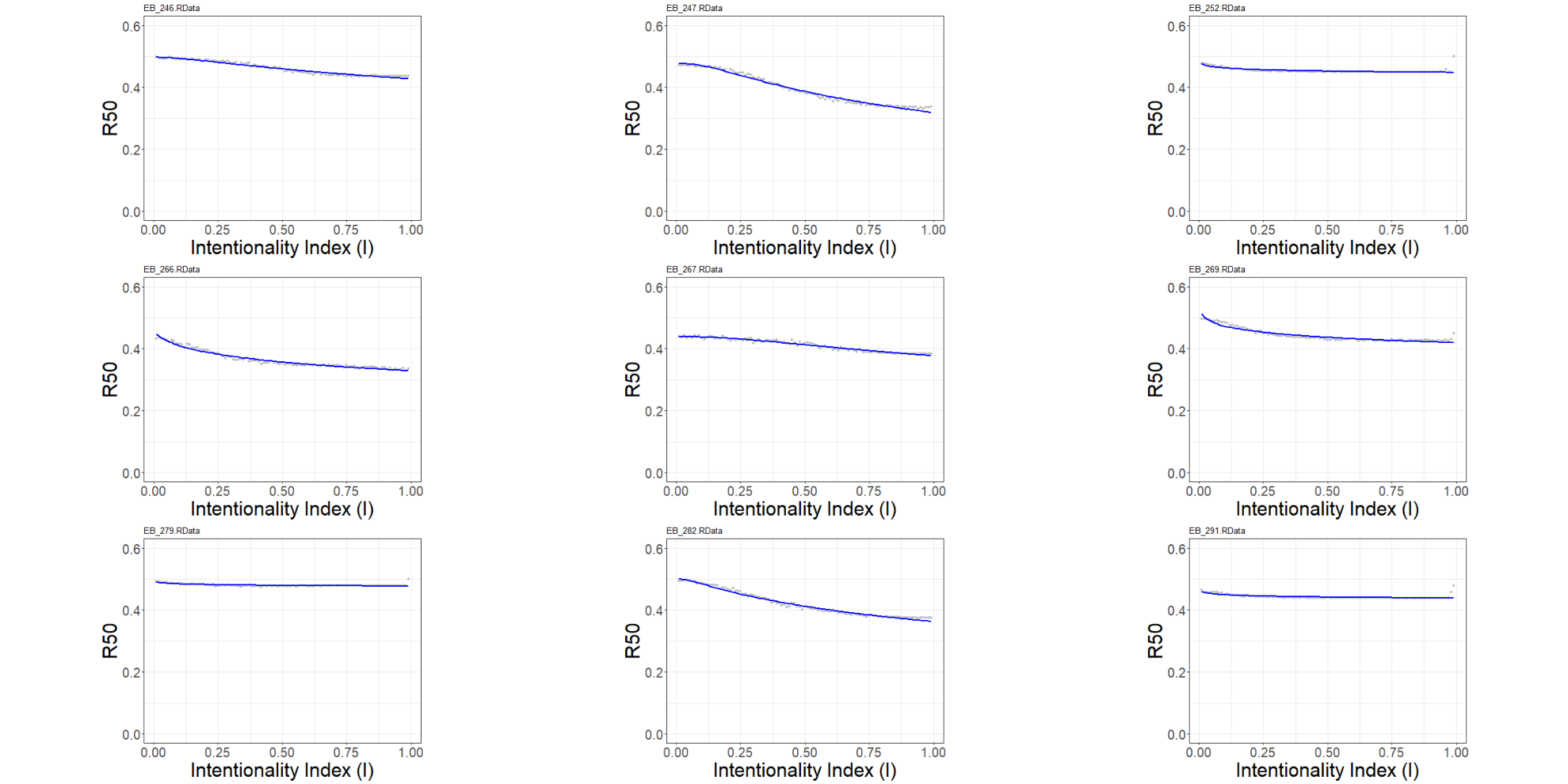

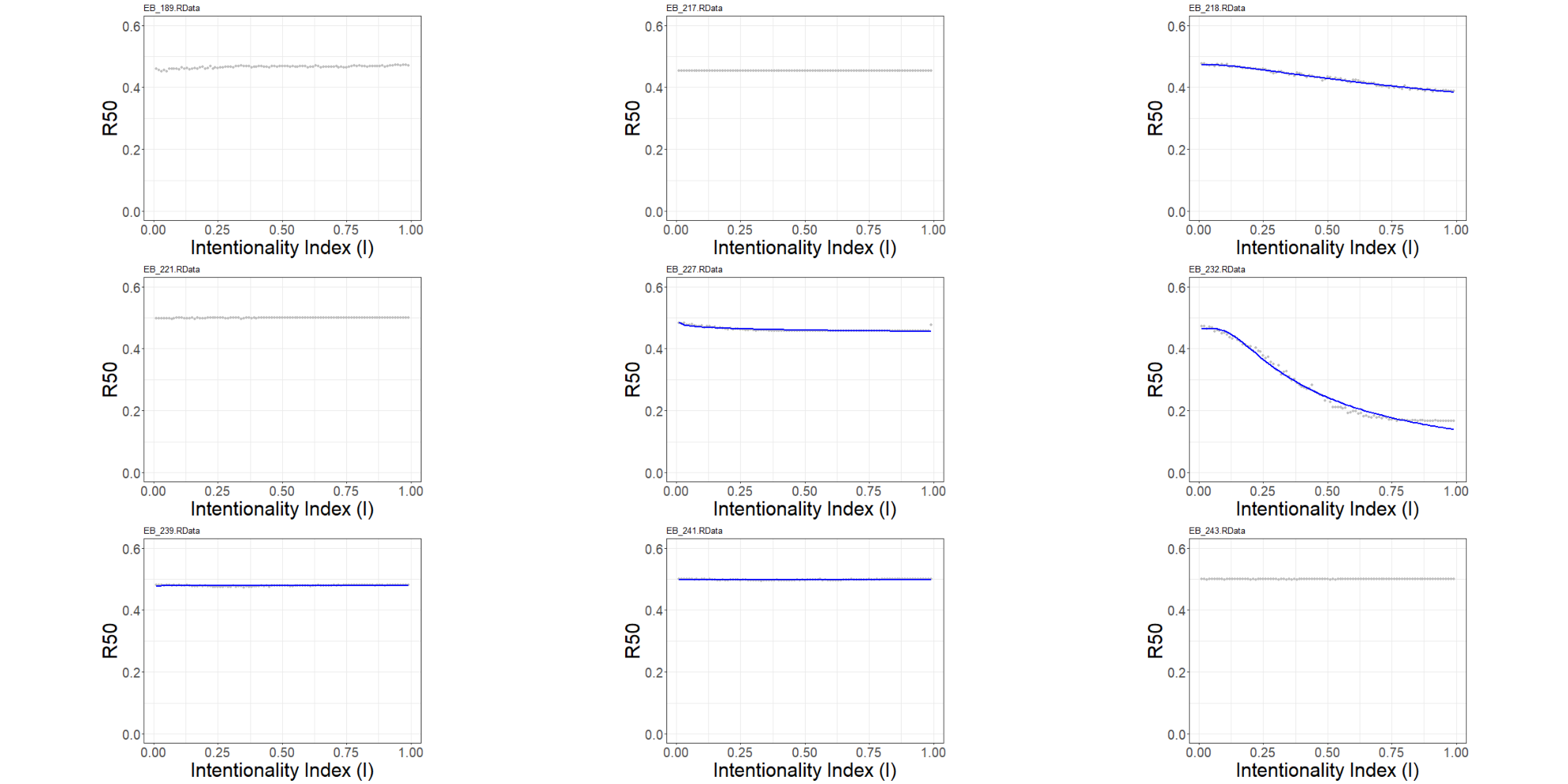

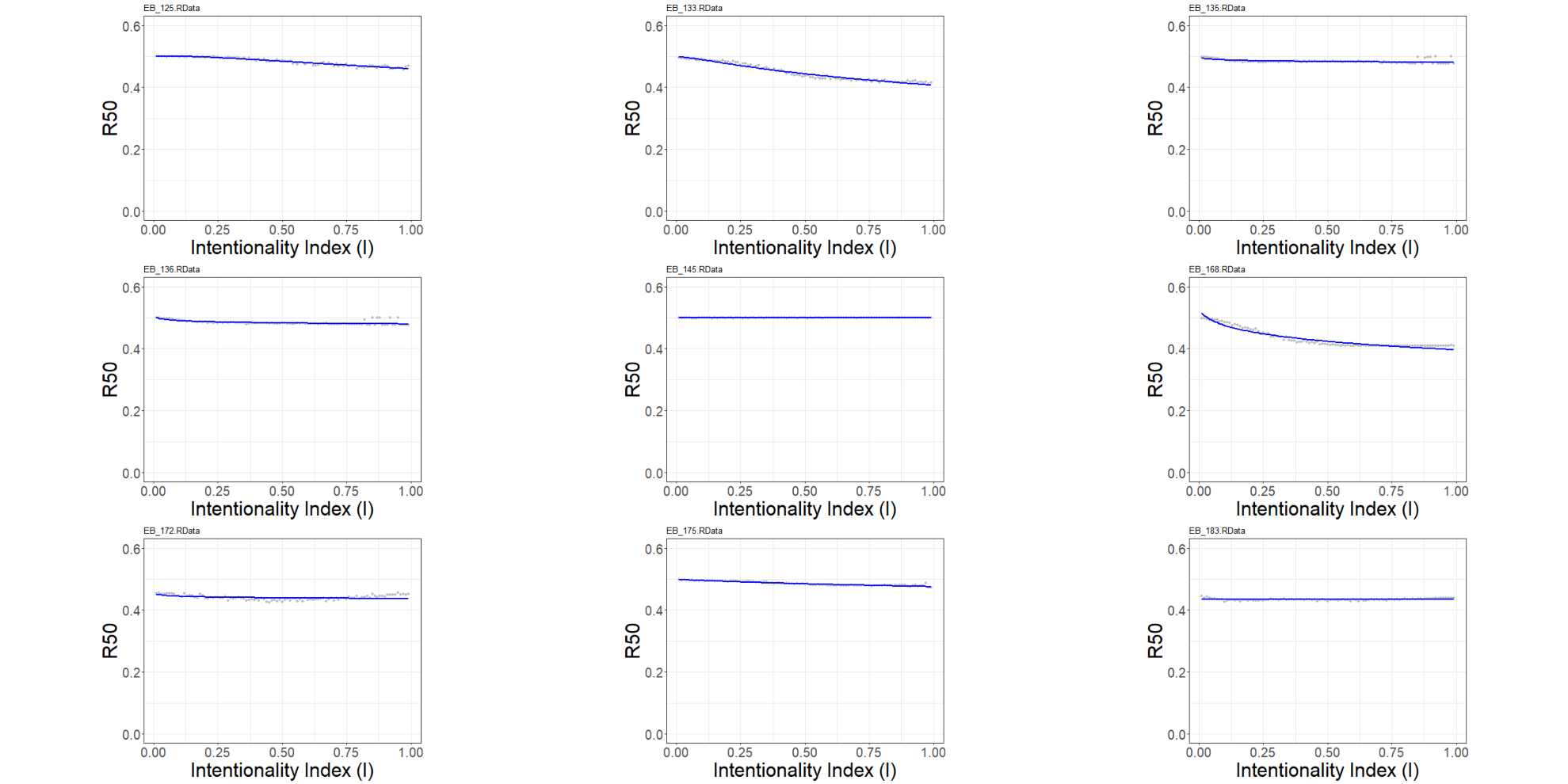

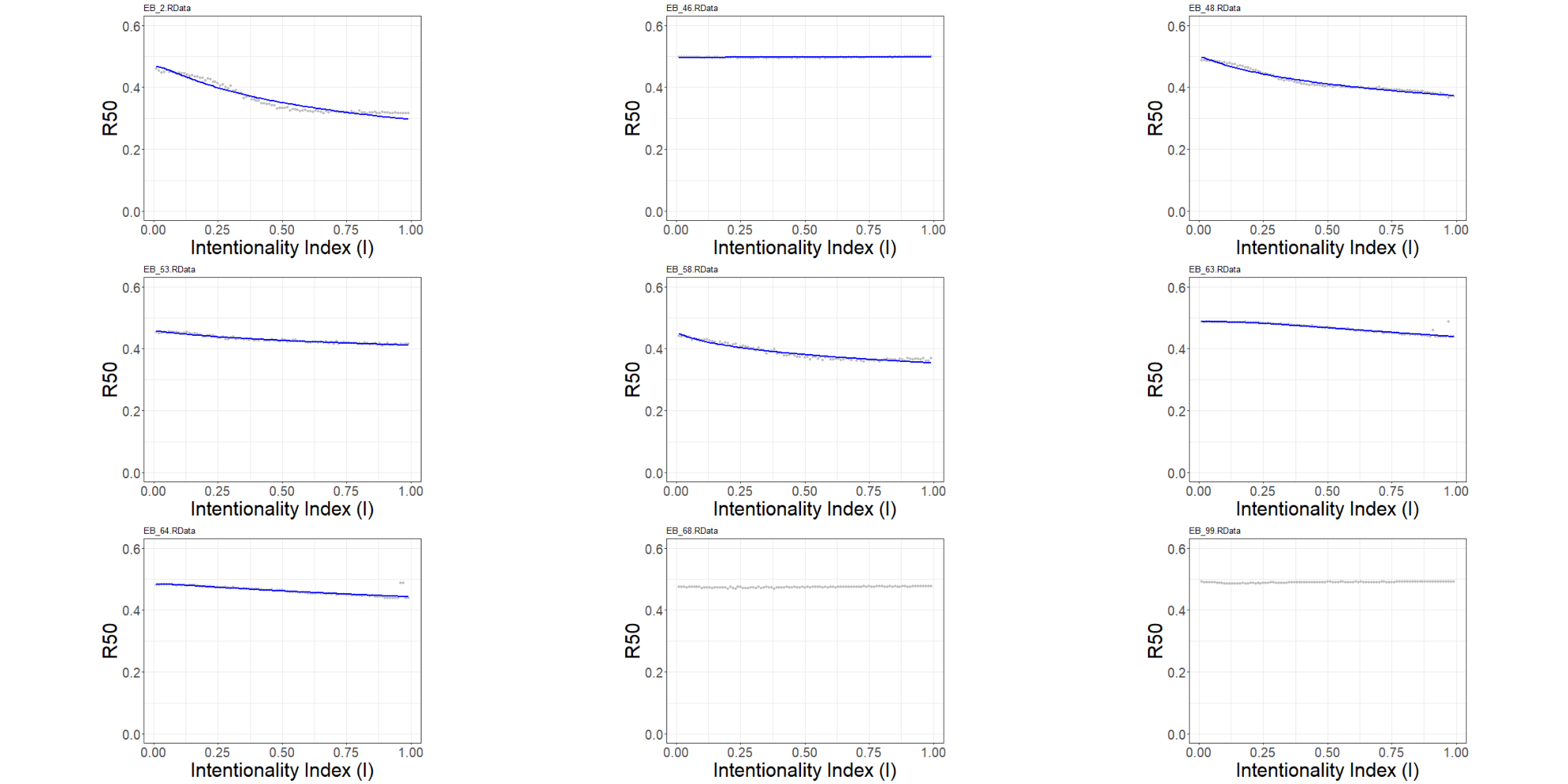

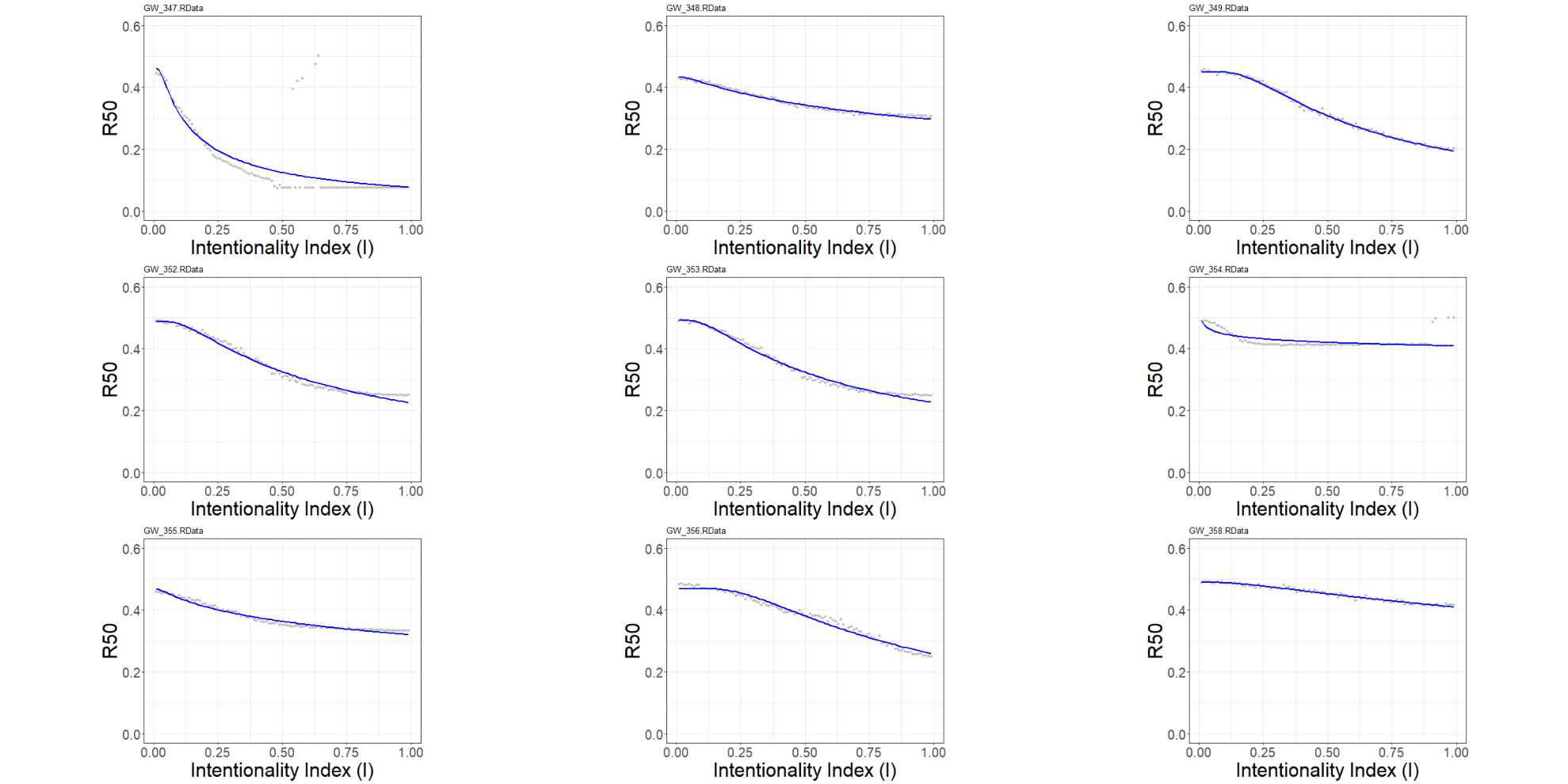

Supplement: Supplementary file 1 — Appendix S1 Appendix S2 [file ELE-25-2476-s001.docx]
